# Force transmission and SUN-KASH higher-order assembly in the LINC complex models

**DOI:** 10.1016/j.bpj.2023.11.001

**Published:** 2023-11-02

**Authors:** Ghafar Yerima, Nya Domkam, Jessica Ornowski, Zeinab Jahed, Mohammad R.K. Mofrad

**Affiliations:** 1 Molecular Cell Biomechanics Laboratory, Departments of Bioengineering and Mechanical Engineering, University of California, Berkeley, California; 2 Department of Nanoengineering, Jacobs School of Engineering, University of California, San Diego, California; 3 Molecular Biophysics and Integrative Bioimaging Division, Lawrence Berkeley National Lab, Berkeley, California

## Abstract

The linkers of the nucleoskeleton and cytoskeleton (LINC) complex comprises Sad-1 and UNC-84 (SUN) and Klarsicht, ANC-1, SYNE homology (KASH) domain proteins, whose conserved interactions provide a physical coupling between the cytoskeleton and the nucleoskeleton, thereby mediating the transfer of physical forces across the nuclear envelope. The LINC complex can perform distinct cellular functions by pairing various KASH domain proteins with the same SUN domain protein. Recent studies have suggested a higher-order assembly of SUN and KASH instead of a more widely accepted linear trimer model for the LINC complex. In the present study, we use molecular dynamics simulations to investigate the mechanism of force transfer across the two proposed models of LINC complex assembly, namely the 3:3 linear trimer model and the 6:6 higher-order model. Employing steered molecular dynamics simulations with various structures using forces at different rates and directions, we examine the structural stability of the two models under various biologically relevant conditions. Our results suggest that both models can withstand and transfer significant levels of force while retaining their structural integrity. However, the force response of various SUN/KASH assemblies depend on the force direction and pulling rates. Slower pulling rates result in higher mean square fluctuations of the 3:3 assembly compared to the fast pulling. Interestingly, the 6:6 assembly tends to provide an additional range of motion flexibility and might be more advantageous to the structural rigidity and pliability of the nuclear envelope. These findings offer insights into how the SUN and KASH proteins maintain the structural integrity of the nuclear membrane.

## Significance

The linkers of nucleoskeleton and cytoskeleton (LINC) complex connects the inner and outer nuclear membranes and transduces force from the cytoplasm to the nucleoplasm. Using molecular dynamics simulations on various structures using forces at different rates and directions, this study aims to examine the mechanics and structural stability of the LINC complex models under various biologically relevant conditions. It provides insights on the mechanisms of force transmission across the nucleus, which may be further investigated via in vitro experiments.

## Introduction

The linkers of nucleoskeleton and cytoskeleton (LINC) complex is a vast network of proteins involved in the mechanical response of the cell (1,2,3). The main components of the LINC complex are the Sad1 and UNC-84 (SUN) and Klarsicht, ANC-1, and SYNE/Nesprin-1 and -2 Homology (KASH) domain proteins (4). SUN proteins are anchored to the inner nuclear membrane and contain small domains in the nucleoplasm and large domains that extend to the outer nuclear membrane. KASH proteins contain a small KASH domain in the perinuclear space and large domains that extend into the cytoplasm (5,6). Both SUN and KASH proteins can transduce forces from the cytoplasm to the nucleoplasm (7,8,9). Of the five different SUN proteins, only two, namely SUN1 and SUN2, are commonly found in virtually all cells (10). KASH domain proteins function not only inside the nuclear envelope but also in the cytoplasm. There are at least six mammalian KASH domain proteins, namely Nesprins 1–4, KASH 5, and lymphoid-restricted membrane proteins (11,12,13,14). Nesprin 1 and 2 are widely expressed in most cell types, whereas Nesprin 3, 4, and KASH5 are cell-type specific. KASH can interact with various cytoskeletal elements: F-actin (15), microtubules (16), intermediate filaments via a plectin binding site (17), and the dynein dynactin (18) complexes, which perform specific cell functions.

Mutations in SUN and KASH proteins are believed to lead to a variety of structural and functional defects in the cell and have been linked to various human diseases (19,20,21,22,23). For instance, Nesprins mutations are associated with autosomal recessive cerebellar ataxia (24), recessive arthrogryposis multiplex congenita (25), hearing loss (26), Meckel-Gruber syndrome (27), depression, and bipolar disorder (28). SUN mutations are associated with DYT1 dystonia (29) and muscular dystrophy, including Emery-Dreifuss muscular dystrophy (30).

**Figure 1:**
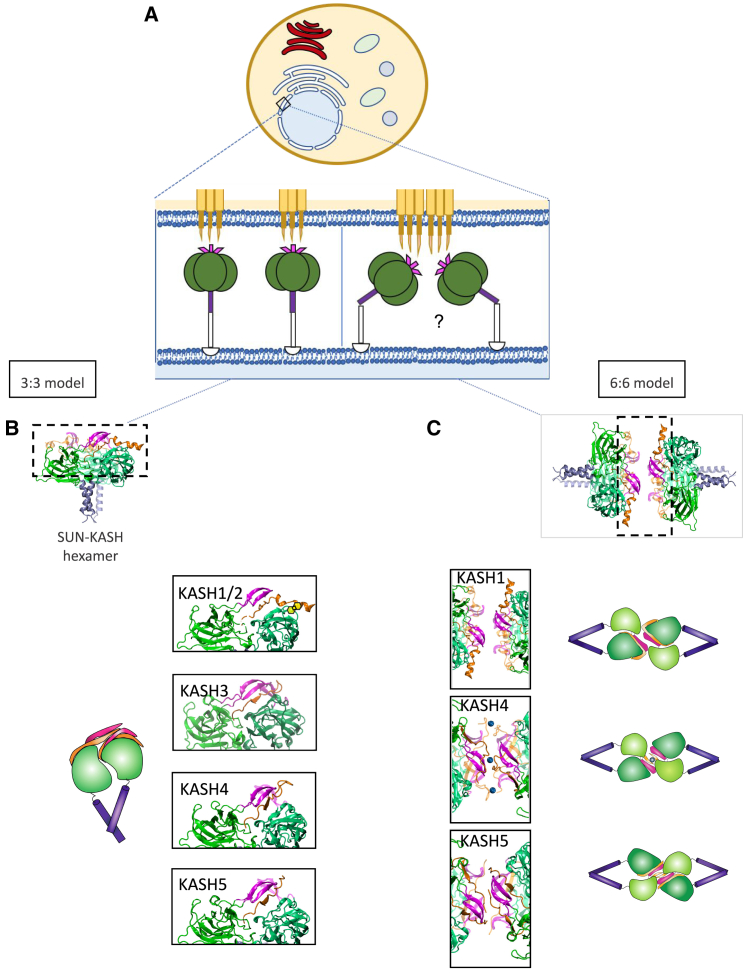
Linear trimer model and higher-order assembly of SUN1,2 in complex with various KASH. (*A*) Schematic of a cell with an enlarged view of the nuclear membrane, which displays the main components of the LINC complex. From top to bottom of the enlarged view, we have the Nesprins and KASH domains (*orange*), the KASH-lids (*pink*), the SUN domains (*green*), and the CCs (*purple*). (*B*) Crystal structure of the linear trimer model or 3:3 model with the different SUN2/KASH pairs. The right column shows the adjacent SUN domains (*green*), the KASH domain (*orange*) as well as the KASH-lids (*pink*) of KASH1,2, KASH3, KASH4, and KASH5. A disulfide bond between the SUN domain and the neighboring KASH is represented in yellow for KASH1,2. (*C*) Crystal structure of the higher-order assembly or 6:6 model with the different SUN1/KASH pairs and an enlarged view of the head-to-head interaction. The left column shows the SUN domains (*green*), the KASH domain (*orange*) as well as the KASH-lids (*pink*) of KASH1, KASH4, and KASH5. To see this figure in color, go online.

Several studies by our group and others have aimed at understanding the molecular mechanisms of force transfer across SUN/KASH complexes through experimental and computational techniques. Notable advancements in our understanding of the LINC complex molecular structure were made by the solved crystal structure of the conserved SUN2/KASH2 interaction by three independent groups a decade ago (5,31,32). The crystal structure revealed an arrangement referred to as the 3:3 model, meaning that each SUN forms a trimer to interact with three KASH, forming an overall hexamer (Figs. 1 *A* and *B* and S1 in the Supporting material). These structural findings suggested that SUN/KASH arrange as linear arrays in the nuclear envelope (31). Studies based on these crystal structures revealed several details regarding the molecular mechanisms of force transfer across the SUN/KASH complex. For instance, recent studies using combined in silico molecular dynamics simulations and in vivo *Caenorhabditis elegans* genetics showed that a mutation of tyrosine at position −7 of KASH disrupts SUN/KASH interaction (32). We showed that a disulfide bond is required for maximal force transmission in SUN2/KASH1,2 (33), and different KASH proteins may transfer distinct magnitudes of force (34). However, all the above-mentioned studies were based on the linear trimer model of SUN/KASH, using the SUN2/KASH1,2 structures. Recently, new crystal structures of the SUN/KASH interaction were released by two groups (35,36). These new structures suggest an alternative model in which SUN/KASH pairs may form higher-order assemblies instead of the putative linear trimer model (35). This alternative arrangement is known as the 6:6, in which two SUN/KASH hexamers (3:3 arrangements) interact through their SUN and KASH domains in a head-to-head fashion and form an overall dodecamer (Figs. 1 *A* and *C* and S1).

**Figure 2:**
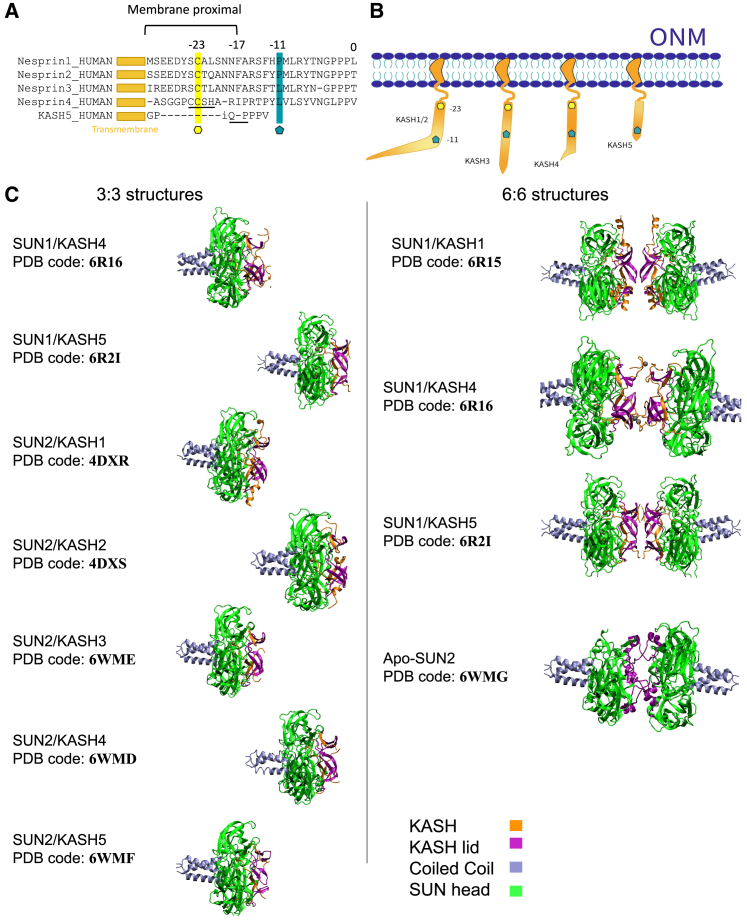
Comparisons between the different structures. (*A*) Multiple sequence alignment of various human KASH domain proteins. Position 0 marks the C terminus of the KASH domains. Position −11 corresponds to a proline in KASH 1/2 and a leucine in KASH 3–5 (*blue pentagon*). Position −23 is occupied by a cysteine residue in KASH 1–4 (*yellow hexagon*). CCSH and PPP domains are found in KASH4 and KASH5 respectively (*underlined*). (*B*) Schematic of the perinuclear space containing KASH domains. A 90° kink at proline −11 is observed in KASH1/2. This kink is not observed in the KASH3–5 structures. (*C*) SUN/KASH structures studied for each model with their corresponding PDB code. The main components are color coded. To see this figure in color, go online.

The 6:6 assembly was solved for three SUN/KASH pairs, namely SUN1/KASH1, SUN1/KASH4, and SUN1/KASH5 (35). Each one of these complexes involves a unique binding modality. In the SUN1/KASH1 complex, the KASH-lid, a region within a SUN protomer, maintains the 6:6 head-to-head interaction. Zinc-cysteine coordination bonds in SUN1/KASH4 maintain the head-to-head interaction. SUN1/KASH5, like SUN1/KASH1, employs the KASH-lid to preserve its head-to-head interaction. The only difference is an additional interaction between the KASH domains of the opposing SUN/KASH hexamers, which is mediated by a PPP motif (35). Additionally, novel 3:3 complexes were crystallized, namely SUN2/KASH3, SUN2/KASH4, and SUN2/KASH5 configurations (36). Unlike KASH1,2 which form a 90° angle due to a proline on the −11th position, the other three KASH do not make a 90° kink and lie along the SUN protomer (Fig. 2 *A* and *B*).

Because force transfer across the LINC complex depends highly on how various LINC complexes assemble in the nuclear envelope, a detailed evaluation and analysis of the two models is needed. To our knowledge, no studies have compared the molecular mechanisms of force transfer across the two models for various SUN/KASH pairs. Moreover, little is known about the response of the SUN/KASH complex to mechanical forces at varying directions and rates, conceivable at various cellular processes.

In this study, we develop all-atom molecular dynamics simulations to analyze and compare the mechanism(s) of force transfer across the two proposed models of LINC complex assembly, namely the 3:3 linear trimer model and the 6:6 higher-order model. Specifically, we investigate SUN2 in complex with KASH1-5 and SUN1 in complex with KASH1,4 and 5. Because of the similarity between SUN1 and SUN2, the structures in both models can be effectively compared based on the KASH type. Thus, we conduct molecular simulations to expose the structures to mechanical forces at different rates and directions and evaluate the structural stability of the two models under various biologically relevant conditions. We used uniaxial pulling for the 3:3 linear trimer and 6:6 higher-order model and transverse pulling for the 6:6 higher-order model. By comparing the force response and conformational changes in each model, our results suggest that both models can withstand high forces without significant deformation. Moreover, the higher-order assembly provides an additional range of motion and might be more suitable for explaining the pliability of the nuclear envelope. Ultimately, these findings offer insight into how the SUN and KASH proteins assemble in the nuclear membrane and maintain the structural integrity of the nuclear envelope.

## Materials and methods

### Models of SUN/KASH complexes

Seven 3:3 and four 6:6 structures were downloaded from the Protein Data Bank (PDB) and used for our simulations (Figs. 2 *C* and S2). The 3:3 structures were SUN1/KASH4 (PDB: 6R16 (35)), SUN1/KASH5 (PDB: 6R2I (35)), SUN2/KASH1 (PDB: 4DXR (31)), SUN2/KASH2 (PDB: 4DXS (31)), SUN2/KASH3 (PDB: 6WME (36)), SUN2/KASH4 (PDB: 6WMD (36)), and SUN2/KASH5 (PDB: 6WMF (36)). The SUN1 structures, which were originally 6:6 structures, were split in half to obtain their 3:3 versions using the Visual Molecular Dynamics (VMD) software. The four 6:6 structures were SUN1/KASH1 (PDB: 6R15 (35)), SUN1/KASH4 (PDB: 6R16), SUN1/KASH5 (PDB: 6R2I), and Apo-SUN2 (PDB: 6WMG (36)). The SUN1 and SUN2 protomers in each structure had similar lengths (195–196 amino acids) with a few missing residues. However, these residues were not near the regions of interest.

### Simulation protocol

All simulations were performed using GROMACS (37) free software with the CHARMM36 (38) force field. The TIP3P water model was used along with neutralizing salt concentrations of 0.15 M KCl and 0.05 M CaCl to mimic the nuclear environment. All structures were minimized at 5000 steps with an energy tolerance of 1000 kJ/mol/nm and equilibrated for roughly 5 ns with a time step of 2 fs. These simulations were run at a constant temperature of 310 K with Berendsen temperature and pressure coupling. Periodic boundary conditions were applied in all three directions. Two different types of pulling simulations were performed on the structures using the isothermal-isobaric ensemble. In total, six different pulling modalities were conducted involving specific pulling rates, pulling direction, and pulling groups. The structures were pulled at a constant velocity of 10 nm/ns for 0.5 ns and another set of simulations were pulled at 1 nm/ns for 5 ns to achieve the same displacement of 5 nm. For all 3:3 structures, the last residues on the different KASH proteins were pulled in the opposite direction of the coiled-coil (CC) region, whereas the end residues of the SUN domains were fixed in all three dimensions. In the 6:6 structure, the nitrogen on one set of coiled regions was pulled in the opposite direction to the CC region. The KASH in the 6:6 structure in a different simulation series was pulled orthogonally in the direction of the CC domain (Table1).

**Table 1:** Simulation breakdown between the different structures in the 3:3 linear model and 6:6 higher-order assembly

	Structures	Pulling modality	Pulling rate	Simulations run
3:3 linear model	SUN1/KASH4 SUN1/KASH5 SUN2/KASH1 SUN2/KASH2 SUN2/KASH3 SUN2/KASH4 SUN2/KASH5	uniaxial on KASH end residue	1 nm/ns for 5 ns + 10 nm/ns for 0.5 ns	3
6:6 higher-order assembly	SUN1/KASH1 SUN1/KASH4 SUN1/KASH5 Apo-SUN2^a^	transverse on KASH end residue + uniaxial on KASH end residue	1 nm/ns for 5 ns + 10 nm/ns for 0.5 ns	3

a Only the uniaxial on KASH end residue modality was used for Apo-SUN2.

### Post-processing/trajectory analyses

#### Root-mean-square deviation

Root-mean-square deviation of the protein backbone was calculated using GROMACS (37) free software and plotted using Xmgrace (39). Each frame in the trajectory was aligned and referenced with the first frame.

#### Root-mean-square fluctuation

Root-mean-square fluctuation (RMSF) per residue was calculated using GROMACS (37) free software after fitting to the first frame of the pulling simulation and plotted using Python (40). For the 3:3 model structures and for each pulling rate, the data for all SUN and KASH protomers of three simulations were averaged.

#### Pulling force quantification

The force graphs were obtained from the input files of each simulation using GROMACS (37) free software and plotted using Python (40). The data for three simulations for each pulling rate were averaged per structure.

#### Interaction energies

The short-range nonbonded interaction energies for the salt bridge residue pairs (K533-E672, D542-R708) in the 3:3 model and KASH-lid residues (671, 673) in the 6:6 model were calculated using GROMACS (37) free software and plotted using Python (40). The salt bridges were categorized as intramolecular (D542-R708) and intermolecular (K533-E672). The data were then concatenated per salt bridge type for each simulation. The data for three simulations were concatenated and density plots were obtained.

#### Hydrogen-bond analysis

Hydrogen-bond analysis was performed using the GROMACS (37) hydrogen-bond analysis tool. The cutoff distance between the donor and acceptor was set to 0.35 nm and the cutoff angle between the hydrogen atom of the donor and acceptor atom was set to 30°.

#### Heatmap

Piecewise distances between residues were calculated between opposing residues on KASH-lids using VMD. The heatmap was created using the positional data and Python (40) library Seaborn.

#### Angle calculation

New conformational angle changes were obtained using VMD’s position tracker over the simulation time and Excel. The alpha carbons of residues 631 and 619 within the CC region were used to create a vector. This vector is meant to represent the central axis of half the SUN/KASH complex. Each half of SUN/KASH has three vectors, which correspond to three *α* helices. All vectors on one half of SUN/KASH were averaged to represent one vector, which serves as the central axis for a SUN/KASH half. The vector calculations were done through Excel.

#### Visualization

All visualizations were done using the VMD software (41).

## Results

Recent structural biological studies have proposed a higher-order assembly model for the LINC complex, casting doubts on the putative mechanism of force transfer within the SUN/KASH models. In this study, we used molecular dynamics simulations contrasting the putative 3:3 SUN/KASH complex model and the new higher-order assembly 6:6 SUN/KASH complex. Both models were subjected to pulling forces at different pulling rates and directions to mimic biologically relevant conditions. These results suggest the assembly of a more profound SUN/KASH network in the nuclear envelope.

### Mechanics of force transfer in the 3:3 SUN/KASH complex

**Figure 3:**
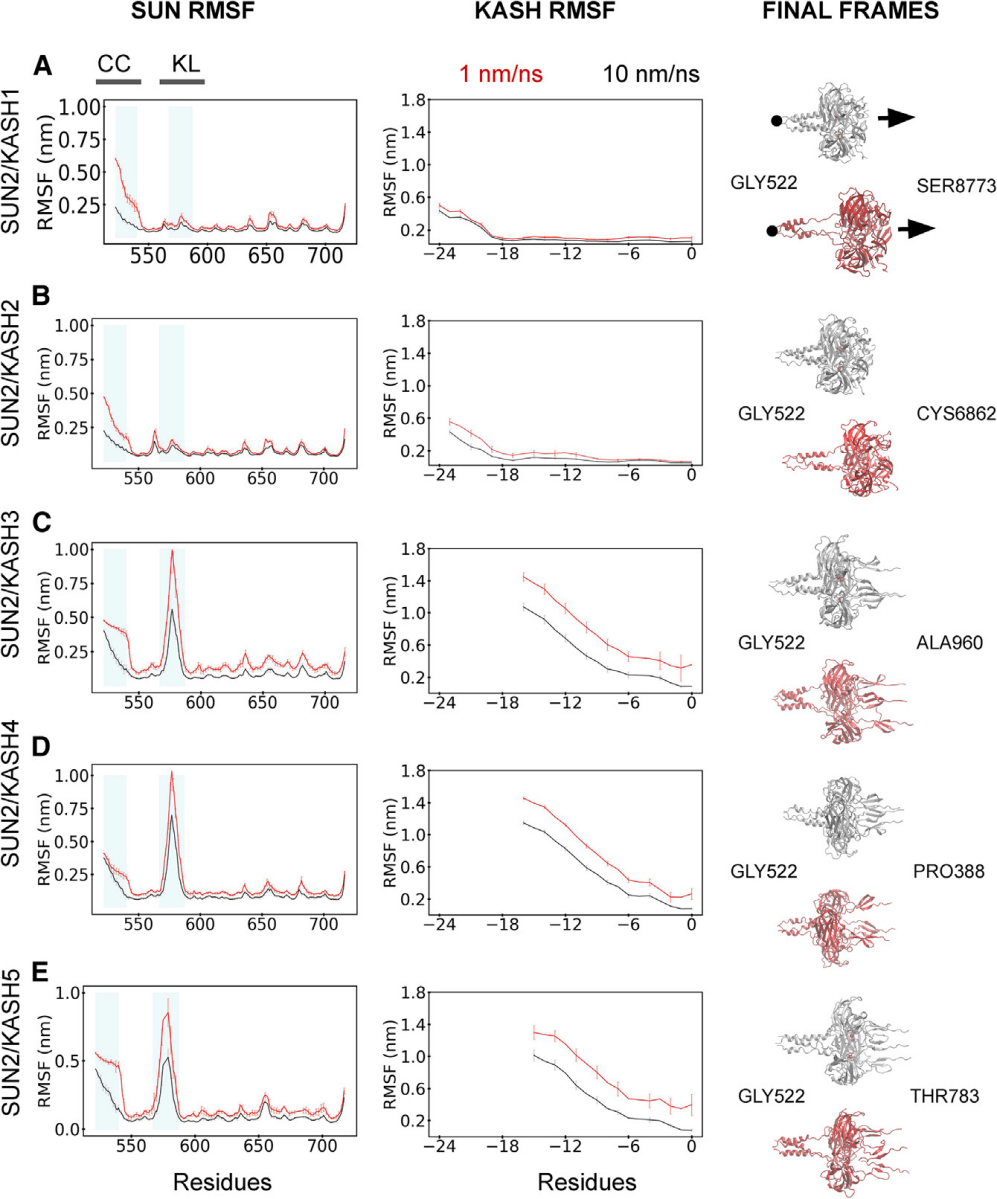
Rate-dependent force response of SUN2 in complex with various KASH. RMSF of SUN2 for (*A*) KASH1, (*B*) KASH2, (*C*) KASH3, (*D*) KASH4, and (*E*) KASH5 is shown in the SUN RMSF (*left*) column. RMSF of the KASH proteins is shown in the KASH RMSF (*middle*) column. The red and black curves represent the 1- and 10-nm/ns pulling rates, respectively. The x axis for the SUN RMSF column graphs ranges from 522 to 716 according to the SUN2 domain residue numbering. The x axis for the KASH RMSF column graph ranges from −24 to 0 following a sequence-alignment-based numbering of KASH proteins (31). CC, coiled-coil domains; KL, KASH-lid. These two areas are shaded in blue on the SUN column graphs. For each graph, the data for three simulations were averaged. For each simulation, the data for all protomers were averaged. The error bars in each plot correspond to the standard deviation of the RMSF of each residue in a protomer of protein domains over all simulations. Both pulling rates reach the same displacement of 5 nm. The final frames of the 10 (*silver*) and 1 (*red*) nm/ns pulling rate simulations are shown for each structure in the right column. To see this figure in color, go online.

The 3:3 SUN/KASH complex was exposed to pulling forces in the uniaxial direction at two rates: 1 nm/ns for 5 ns and 10 nm/ns for 0.5 ns. The last frames of the simulations and the pulling direction are shown in the rightmost column of Fig. 3. In the following, we present the main findings regarding the stability of the 3:3 model.

#### The slow pulling reveals more fluctuation in the CC region in linear 3:3 SUN/KASH assemblies

To determine the structural fluctuation of SUN and KASH under force, we calculated the RMSF of SUN2 and KASH1-5 in SUN/KASH complexes under loads (Fig. 3). Two pulling rates (1 nm/ns and 10 nm/ns) were used to achieve the same displacement of 5 nm. Three simulations were conducted for each pulling rate and each SUN/KASH pair. The results from the three simulations were averaged for each pair. A representative image of the final frames of the simulations is shown on the rightmost column of Fig. 3, displaying the final state of the SUN/KASH complexes after force application. The RMSF plots indicated that SUN2/KASH3–5 exhibit higher fluctuation in the KASH-lid region compared to SUN2/KASH1 and SUN2/KASH2. This is likely due to the proximity of the KASH-lids of SUN2/KASH3, SUN2/KASH4, and SUN2/KASH5 to the KASH domain where the loads are applied. However, both SUN2/KASH1 and SUN2/KASH2 have KASH domains that are further away from the KASH-lid. We also observed less fluctuation in the KASH domain of SUN2/KASH1 and SUN2/KASH2 compared to SUN2/KASH3–5. For all structures, the 1-nm/ns pulling rate shows more fluctuations in the CC domain than the 10-nm/ns rate, suggesting that forces are more likely to transfer to the CC domain in the slow pulling. Moreover, the residues of CC regions for the 10-nm/ns pulling rate show a steady decrease in RMSF, whereas the fluctuation difference over those residues is smaller for the 1-nm/ns rate (Fig. 3). This may suggest that, during the fast pulling, the forces on KASH are rapidly transferred along the protein to the end residue of the CC domain without significant changes in the structure of the protein fragment included in our simulations. However, all CC residues experience an even distribution of stress during the slow pulling. The 3:3 versions of SUN1/KASH4 and SUN1/KASH5, obtained by splitting their 6:6 structures, were also investigated (Fig. S3). The RMSF distribution of the 3:3 form of SUN1 in complex with KASH4 was similar to SUN2/KASH1 and SUN2/KASH2, whereas the SUN1/KASH5 RMSF resembles the other three structures. The reason for this stems from KASH4 in the split version being longer (23 residues) than KASH4 (17 residues) in the SUN2 original structures. Thus, the KASH-lid region is further away from the end KASH residue.

#### SUN2/KASH1 and SUN2/KASH2 can withstand higher forces than the other SUN/KASH pairs in the 3:3 linear model

**Figure 4:**
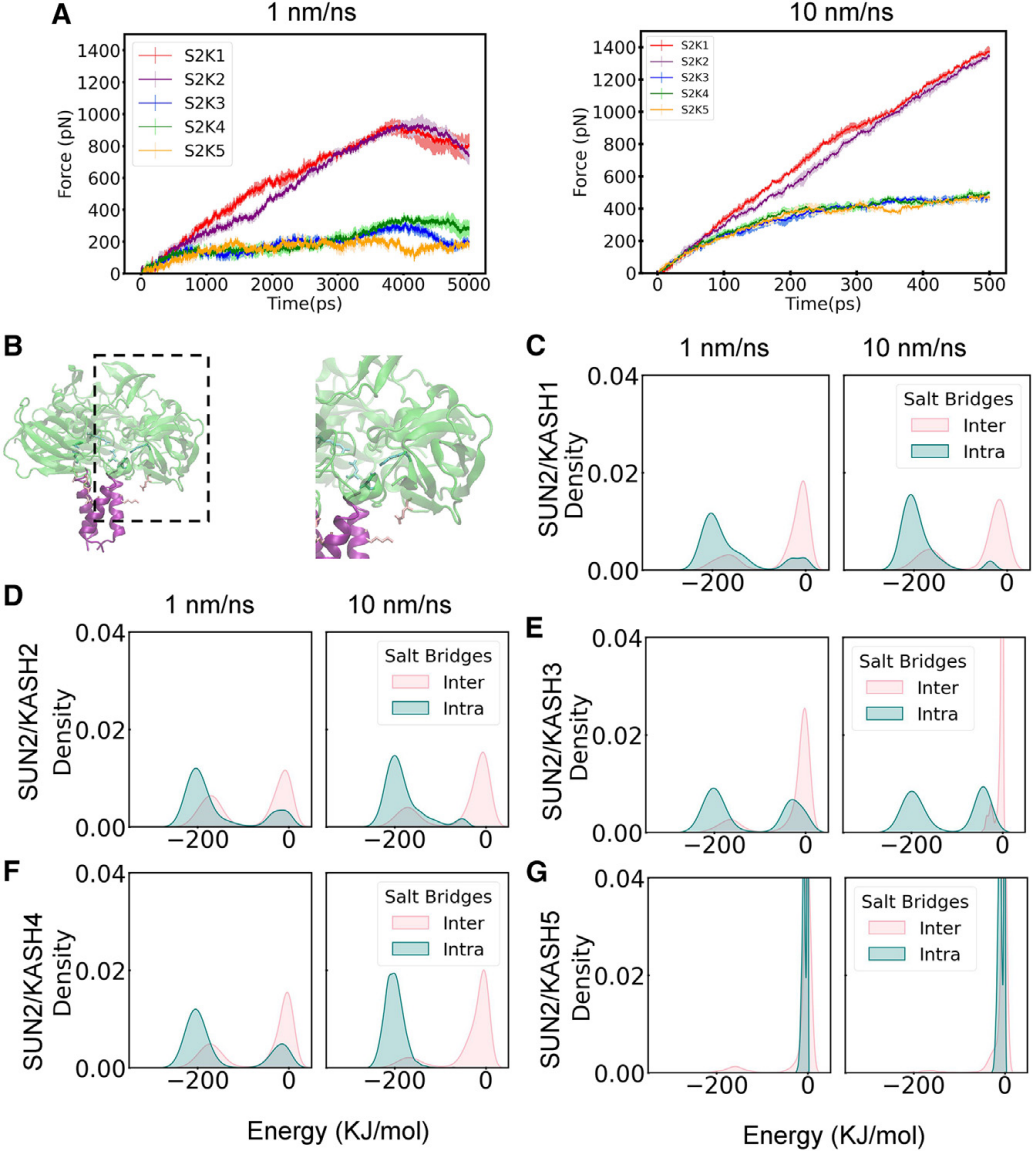
Pulling force and salt bridge interaction energies of SUN2 in complex with various KASH. (*A*) Pulling force in pN over time of SUN2/KASH1 (*red*), SUN2/KASH2 (*purple*), SUN2/KASH3 (*blue*), SUN2/KASH4 (*green*), and SUN2/KASH5 (*brown*). The left graph represents the 1-nm/ns pulling rate and the right graph shows the 10-nm/ns one. The x axis ranges from 0 to 5000 ps for the 1-nm/ns rate and from 0 to 500 ps for the 10-nm/ns rate following the simulation time. Each simulation ran to achieve the same displacement of 5 nm. For each pulling rate and structure, three simulation data were averaged. The error bars on both graphs correspond to the standard deviation of force output over all three simulations. (*B*) Inter salt bridges (*pink*) between residues K533 of CC region and E672 of the neighboring SUN domain, and intra salt bridges (*cyan*) between residues D542 and R708 of each SUN protomer. The three inter and three intra salt bridges data were concatenated per type for each simulation. The y axis shows the density and the x axis represents the interaction energies in kJ/mol. Density plots of salt bridge interaction energies of SUN2 with (*C*) KASH1, (*D*) KASH2, (*E*) KASH3, (*F*) KASH4, and (*G*) KASH5. For each figure, the left graph shows the 1-nm/ns rate and the right graph represents the 10-nm/ns one. To see this figure in color, go online.

To further investigate the reasons for the differences in RMSF, we looked at the force required to pull the various complexes apart over the trajectory of the simulation (Fig. 4 *A*). For both simulation rates, a larger force was required to pull SUN2/KASH1 and SUN2/KASH2 compared to the other three complexes. The force at the end of the shorter simulation is around 1300 pN for KASH1 and KASH2 and ∼400 pN for the other three structures. On the other hand, the 5-ns simulation at 1 nm/ns revealed a maximum force of ∼900 pN for SUN2/KASH1 and KASH2 and ∼330 pN for SUN2/KASH3–5. The force discrepancy confirms previous findings that SUN2 in a complex with KASH1 and KASH2 can withstand higher forces than KASH3, KASH4, and KASH5 (33). SUN2/KASH1 and SUN2/KASH2 exhibit a linear elastic spring behavior throughout the 10-nm/ns simulation with a mostly linearly increasing force, whereas the force for SUN2/KASH3–5 increases at a significantly slower rate. We notice a drop in the force intensity around 4 ns for all structures during the longer simulations. The decrease in force is more pronounced for SUN2/KASH5 compared to SUN2/KASH3 and SUN2/KASH4, which are more alike. We further investigated this force drop in the following section.

#### Slower pulling rates result in rapid breakage of conserved intramolecular salt bridges of SUN2 in the linear SUN/KASH model

To determine the reason for a sudden drop in forces, we identified crucial residue interactions that contribute to the stability of the SUN trimer under force. We specifically looked at two important salt bridges, one linking the SUN domain of each SUN protomer to the *α* helix (in the CC region) of the neighboring protomer (inter) and another within the SUN domain itself (intra) (Fig. 4 *B*). We calculated the interaction energies between residues K533 and E672 that form the intermolecular salt bridge and between D542 and R708 that form the intramolecular salt bridge (Fig. S4). The salt bridge observations were concatenated per type (i.e., inter- vs. intramolecular) for each structure and simulation, and the resulting data were used to obtain density plots (Fig. 4 *C*–*G*). The main difference between the two simulation rates for all structures except SUN2/KASH5 is that all salt bridges break for the longer simulation, whereas some of them remain unbroken for the shorter (Fig. S4). All the inter salt bridges tend to break earlier than intra salt bridges as shown by their null interaction energies. This can better be seen on the density plots, where the density for the inter salt bridges around 0 kJ/mol is generally greater than the intra salt bridges and they decrease in density with a decrease in pulling speed at the same energy (Fig. 4). Most intra salt bridges tend to break during the longer simulations, whereas they do not break during the shorter simulations. The broken salt bridges can be seen by a higher intra density ∼0 kJ/mol for the longer simulation as compared to the shorter one. For the SUN2 KASH5 complex, most of the interactions break earlier over the course of the simulation. The short-lasting salt bridges are likely due to KASH5 being the shorter KASH protomer.

### Mechanics of force transfer in the 6:6 SUN/KASH complex

**Figure 5:**
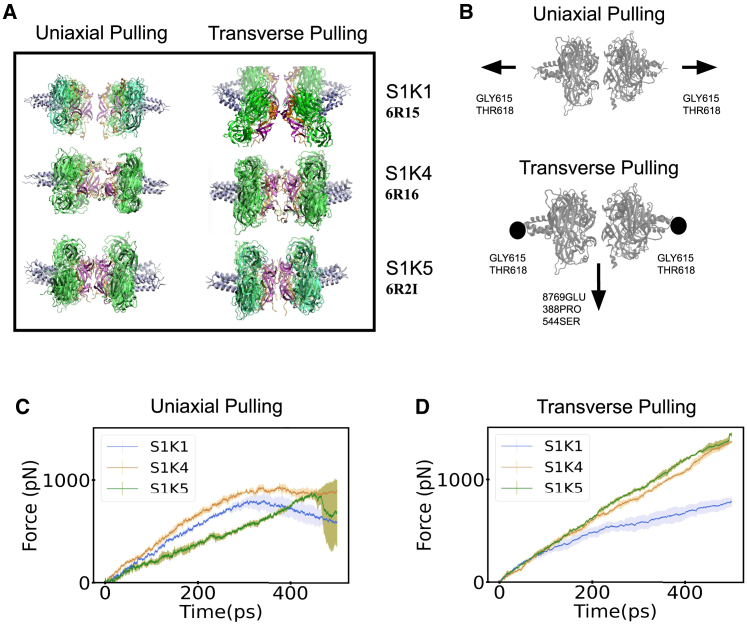
Higher-order assembly model pulling modalities and forces. (*A*) First and last frame of the SUN/KASH 6:6 structures for both pulling modalities. Each is color coded to show the different regions of the protein. The pink shaded regions represent the KL within each structure. The orange shaded proteins are the KASH domains. The green shaded region is the SUN domain, and the gold shaded regions are KASH domain proteins. (*B*) Depiction of uniaxial and transverse pulling. (*C* and *D*) A comparison of the amount of force used throughout the simulation for each pulling modality over the simulation time. The error bars in these plots correspond to the standard deviation of the force output over all simulations. To see this figure in color, go online.

In the next sections, we consider the main results regarding the stability of the 6:6 or higher-order assembly of the SUN/KASH complex (Fig. 5). The main components of the different higher-order assembly complexes are shown in Fig. 5 *A*. We pulled on the 6:6 complex in two different directions: uniaxial and transverse (Fig. 5 *A* and *B*). We also performed uniaxial pulling on the Apo-SUN2 structure (Figs. S5 and S6). We will present the observed differences between each pulling direction and expose our justification for these force directions by discussing some potential biological processes where SUN/KASH could possibly experience these forces.

#### SUN/KASH hexamers completely dissociate in SUN1/KASH1 under uniaxial pulling (but not in SUN1/KASH4 and SUN1/KASH5)

Contrasting the initial and final frames of the molecular dynamics trajectory of the uniaxial pulling simulation, we can infer that the separation of KASH heads for SUN1/KASH1 is more appreciable than for SUN1/KASH4 and SUN1/KASH5 (Fig. 5 *A*). Pulling the SUN/KASH heads apart requires the same force magnitude until 0.3 ns (Fig. 5 *C*). At 0.3 ns of the simulation, the SUN1/KASH1 trimers completely separate, whereas SUN1/KASH4 and SUN1/KASH5 still maintain some of their head-to-head interactions. The lack of complete dissociation is shown in the uniaxial pulling column of Fig. 5 *A*, where there are clear differences between the distances separating KASH heads. The structural differences between the SUN/KASH systems (Fig. 1 *C*) are responsible for discrepancies in separation. The same could be said for slower pulling rates. The amount of force required to break the head-to-head interaction is smaller, but the overall trends remain the same. The next pulling modality we looked at is transverse pulling.

#### SUN1/KASH1 experiences a more distinct conformational change than SUN1/KASH4 and SUN1/KASH5 but less force under transverse pulling

**Figure 6:**
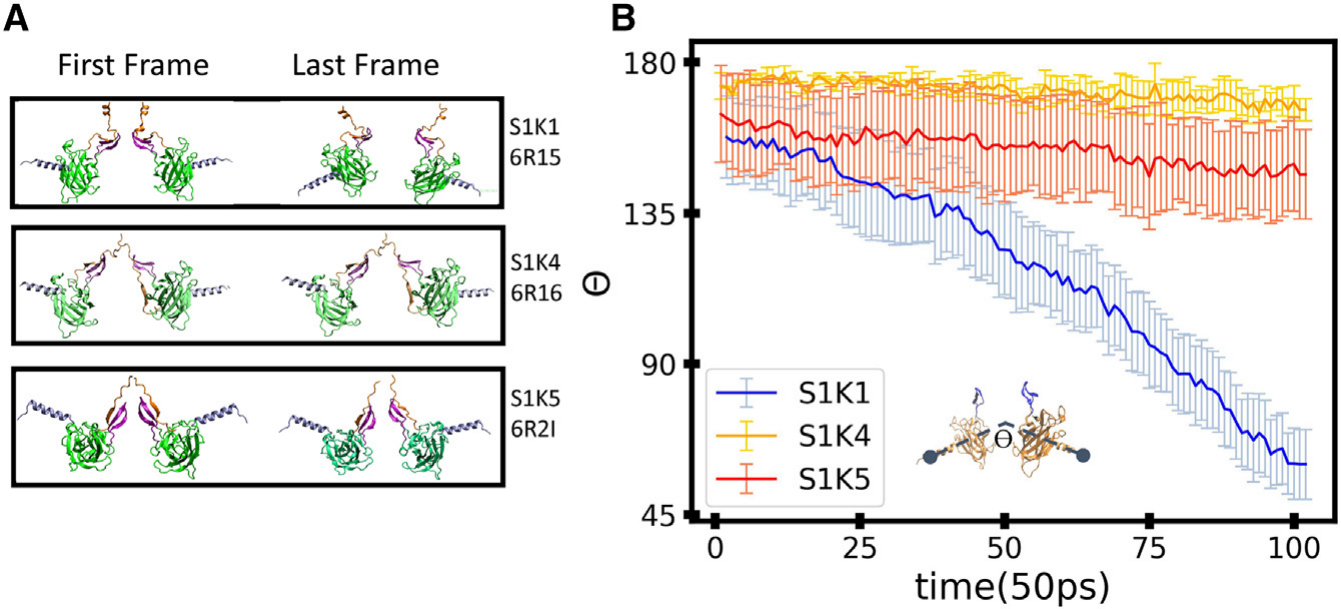
Conformational changes within SUN/KASH structures under transverse pulling. (*A*) The first and last frames of transverse pulling for all KASH containing 6:6 structures. Only two protomers were used to show the change in conformation. (*B*) Angle changes between *α* helices of adjacent SUN protomers over the simulation time (*Ө*) as shown in the inset schematic. To see this figure in color, go online.

In the transverse pulling modality, we applied forces to the end of each KASH instead of SUN. The transverse pulling modality showed some distinct conformational changes within the different SUN/KASH structures (Fig. 6 *A*). The force required to pull on KASH was noticeably greater in SUN1/KASH4 and SUN1/KASH5 as compared to SUN1/KASH1 (Fig. 5 *D*). The SUN1/KASH1 structure reaches a maximum load of 787 pN under transverse pulling, whereas SUN1/KASH4 and SUN1/KASH5 attain a maximum force of 1364 and 1438 pN, respectively, for transverse pulling (Fig. 5 *D*). The differences in maximum force between SUN1/KASH1 and SUN1/KASH4,5 are directly related to the head-to-head interaction. However, before we examine the head-to-head interaction between the different structures, we should quantify the structural changes experienced by each SUN/KASH complex. The change in angle between CC regions on opposing SUN trimers was used to calculate the overall angle change of the structure. A simplified version of all 6:6 structures in the first and last frames of transverse pulling and the rate of angle change over the simulation time are presented in Fig. 6 *A* and *B*. SUN1/KASH1 shows the greatest angle change, whereas, interestingly, SUN1/KASH4,5 show relatively the same angle change over the simulation time.

One may expect the SUN/KASH system to start at roughly 180°; however, in their original structures, the opposing CC regions are not coaxial. SUN1/KASH1 changes 40° over the simulation time. On the other hand, SUN1/KASH4,5 change ∼5° over the simulation time. Like the other results regarding the 6:6 structure, the angle change can be explained by the differences in head-to-head interaction of the three structures.

**Figure 7:**
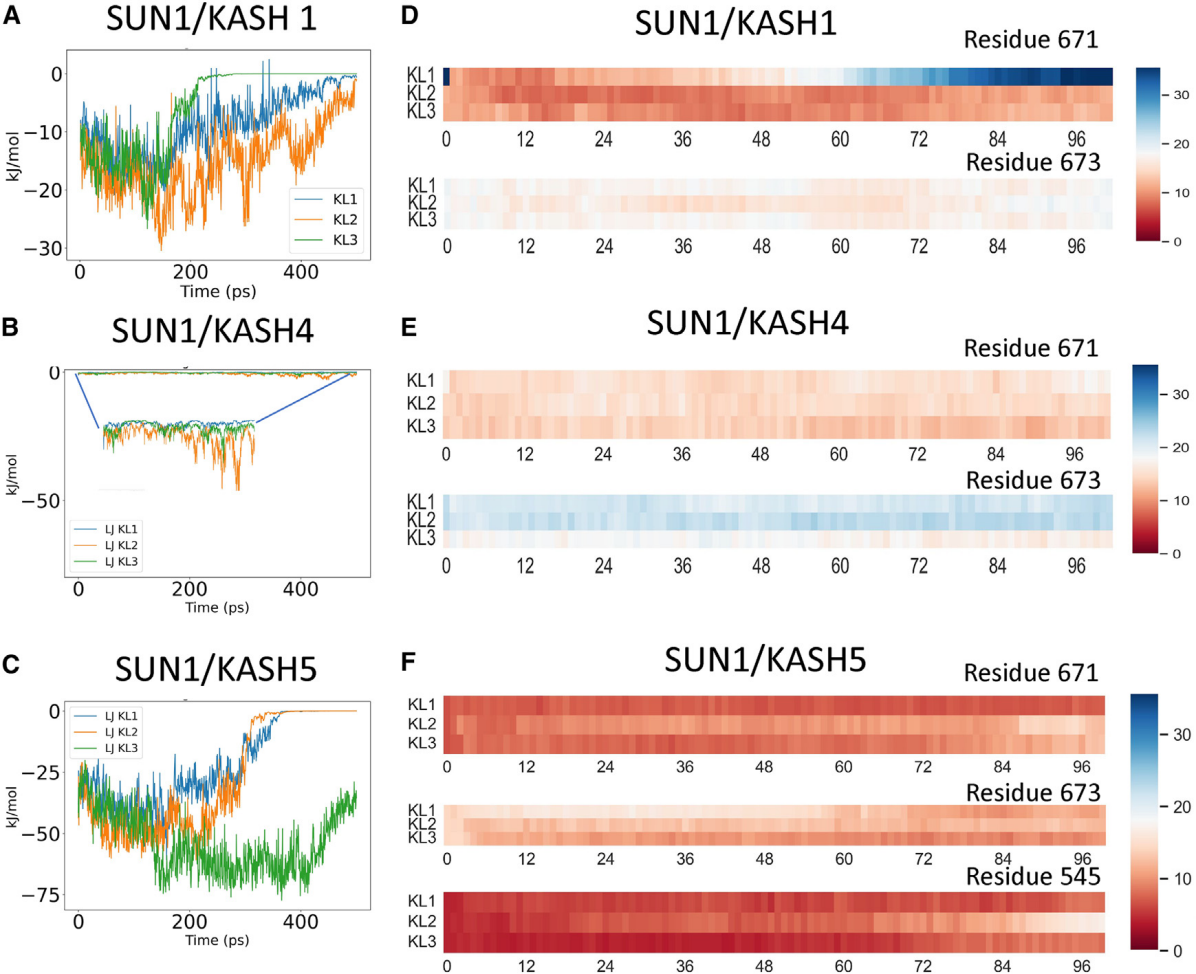
Interaction energies between KLs of various SUN/KASH pairs at 10 nm/ns. (*A*) The Lenard-Jones (LJ) interaction energies between KL pairs (i.e., KL of a protomer on one SUN trimer with adjacent KL of a protomer on other SUN trimer) for (*A*) SUN1/KASH1 (*B*) SUN1/KASH4, and (*C*) SUN1/KASH5. (*D*) Heatmaps of the pairwise distances between the alpha carbons of residues 671 or 673 of adjacent KLs on opposite SUN trimers over the simulation time for SUN1/KASH1 and (*E*) SUN1/KASH4. (*F*) Heatmaps of the pairwise distances between the alpha carbons of residues 671, 673, and 545 of adjacent KLs on opposite SUN trimers over the simulation time for SUN1/KASH5. The color bars of the heatmaps are the distance between residues in angstroms. To see this figure in color, go online.

To determine whether the head-to-head interactions play a role in maintaining the integrity of the structures, we used both GROMACS interaction energies and a pairwise distance heatmap (Fig. 7). We focused on two residues, 671 and 673, on the KASH-lid because these residues are thought to primarily maintain head-to-head KASH-lid interaction. For SUN1/KASH1, there was a steady drop in interaction energies between KASH-lids under force (Fig. 7 *A*). The sudden drop of energy corresponds to the same pairwise distance between residue 671 of corresponding KASH-lids (Fig. 7 *D*). In SUN1/KASH4, the KASH-lid does not participate in bonding; this can be seen on the heatmap (Fig. 7 *E*), and the near-zero interaction energies shown in Fig. 7 *B*. In the case of SUN1/KASH4, the main interactions that hold the structure together are the zinc coordination bonds with KASH. Finally, Fig. 7 *C* shows the different interactions between the various KASH-lid pairs in SUN1/KASH5. In SUN1/KASH5, a breakage of the interaction between residues 545, which corresponds to the PPP motifs, is observed as evident from the heatmap in Fig. 7 *F*.

#### Apo-SUN2 main head-to-head interaction requires considerably less force to break

As control simulation, the force response and interactions within the Apo-SUN2 structure were examined next. Apo-SUN2 refers to the trimer of SUN2 containing a KASH-lid region but no KASH associated. Since both SUN1 and SUN2 have very similar structures, we assumed that the forces experienced and the residues that contribute to the head-to-head interactions are similar. We first decided to compare the amount of force required to split the structure apart (Fig. S5). The Apo structure requires considerably less force to break the head-to-head interaction than the SUN/KASH complexes. This result is consistent across the different pulling rates. Additionally, we considered the interaction energies between the KASH-lids of the different Apo heads. We measured the Lennard-Jones short-range interaction energies between the opposing KASH-lids, given the important role they play in SUN1/KASH1 head-to-head interaction. Our results suggest the KASH-lid also plays a significant role in maintaining the Apo head-to-head interaction (Fig. S5).

### Force and RMSF comparison of the 3:3 and the 6:6 models

The force response and the RMSF were used to compare the uniaxial pulling modality of the linear trimer model and the transverse pulling modality of the higher-order assembly. We believe comparing both models is appropriate, since we are effectively pulling on the KASH protomers in these two modalities. The force over the simulation time between the two models differs greatly. We compared the structures in the linear trimer model to their counterparts in the higher-order assembly based on the KASH. This comparison is motivated by the sequence and structural similarity between SUN1 and SUN2 (42,43,44). Thus, SUN2/KASH1 (3:3 model) experiences a lot more force compared to the 6:6 assembly of SUN1/KASH1 for the same displacement. On the other hand, SUN2/KASH4,5 experience less force in the linear trimer form than the 6:6 model of SUN1/KASH4,5. Both of these phenomena can be explained through the structure of the different SUN/KASH variations and pulling directions.

**Figure 8:**
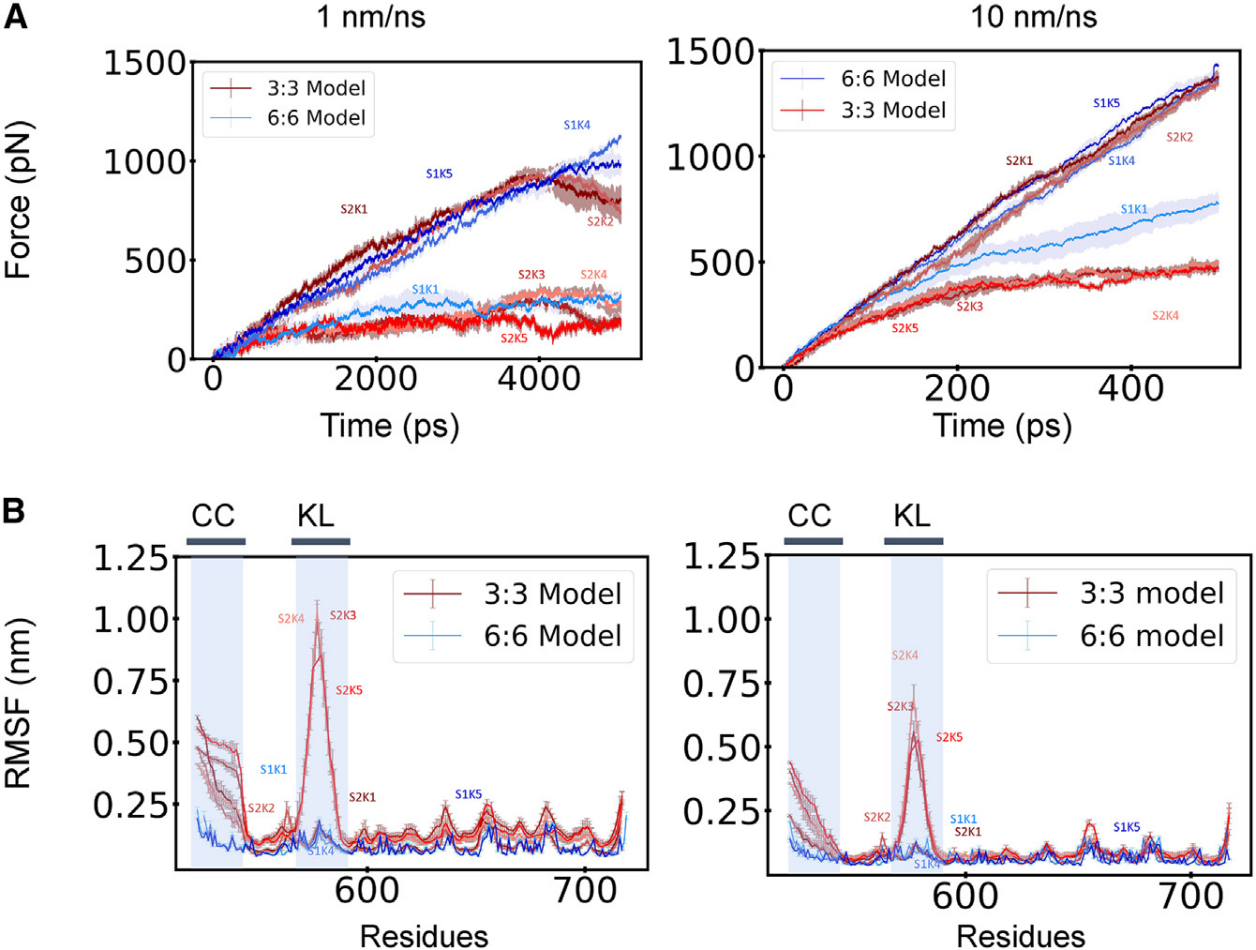
Force and RMSF comparison between the two different models of SUN/KASH over the simulation time. The mode of pulling used to compare for the 6:6 structure is transverse pulling. (*A*) The 6:6 structures are all colored blue, whereas the 3:3 structures are colored red. The SE is shaded in a lighter hue. Unlike 3:3, the SE in 6:6 noticeably increases. With the exception of SUN2/KASH1,2, the 6:6 structures (*blue*) experience more force than the 3:3 structures. The differences between 3:3 and 6:6 are the increased size of the SE and the noticeable decrease in force in the latter half of the simulation. The forces in 3:3 all tend to decrease during the end of the simulation and the forces in 6:6 tend to increase over the simulation time. The error bars in (*A*) corresponds to the standard deviation of the force output over all simulations. (*B*) All of the 6:6 structures are colored in blue, whereas all of the 3:3 structures are colored in red. The residues for 6:6 (contained SUN1) were shifted to match residue numbering in 3:3 (SUN2). The main differences between the two arrangements are the CC region and the KL region. The 3:3 structures have a noticeable peak for SUN2/KASH3,4,5 and a lower peak for all other structures. 3:3 has higher fluctuations in the CC region compared to 6:6. The error bars in the each plot corresponds to the standard deviation of the RMSF of each residue in a protomer of protein domain for all simulations. To see this figure in color, go online.

We also compared the RMSF between the different models. We aligned SUN1 residue numbering to SUN2, following other previous studies on comparing the similarities between SUN1 and SUN2 (44). As seen in Fig. 8, the CC region, which is between 522 and 540, experiences a greater fluctuation in the 3:3 model than in the 6:6 one. The other noticeable change occurs in the KASH-lid region. SUN2/KASH3–5 show large RMSF values in residues 567–587 (KASH-lid region in SUN2), whereas SUN2/KASH1,2, and SUN1/KASH1,4,5 do not exhibit appreciable structural fluctuations. The CC region is closely connected, in terms of residue spacing to the KASH-lid region. Also, by pulling on KASH, we are effectively pulling on the KASH-lid. Consequently, the CC region affects the KASH-lid and vice versa. Overall, SUN1/KASH1,4,5 and SUN2/KASH1,2 do not possess high RMSF compared to SUN2/KASH3–5. In the case of SUN2/KASH1,2, this difference can be explained by the presence of disulfide bonds, which prevent high fluctuations in KASH-lid and CC regions, thereby maintaining the structural integrity of the complexes under high load. For SUN1/KASH1,4,5, other interactions are keeping the SUN/KASH higher-order assembly relatively stable as KASH is pulled in a different direction.

### Hydrogen-bond analysis between KASH and SUN in both the 3:3 and the 6:6 models

Hydrogen-bond analysis was performed on 1-nm/ns trajectories to better understand the qualitative observations in the various trajectories. We decided to focus on two different groups, the hydrogen bonds that form between KASH-lid (residues 661–679 or residues 572–584) and KASH as well as the hydrogen bonds between KASH and SUN (residue 651, 654 or 556, 557). The residues between KASH-lid and KASH form an antiparallel three-strand *β* sheet. We chose to focus on hydrogen bonds because we were interested to see how the interaction between KASH and KASH-lid may change under loads. The best way to understand this is through hydrogen bonds that form between *β* sheets. Throughout all trajectories, the antiparallel *β* sheets between KASH-lid and KASH do not undergo significant visual changes. This observation can be further quantified through kernel density estimate plots of the number of hydrogen bonds between the KASH-lid and KASH (Fig. S7). Both 3:3 and 6:6 structures slightly differ in the number of hydrogen bonds that form; however, the number of hydrogen bonds remains consistent outside of one or two hydrogen bonds over the entire simulation. This result corresponds to not observing changes to the antiparallel *β* sheet region. The differences between the kernel density estimation plots of SUN2/KASH5 and the rest of the 3:3 structures can be explained by missing residues in the KASH-lid. These missing residues would increase the amount of hydrogen bonds observed in SUN2/KASH5.

**Figure 9:**
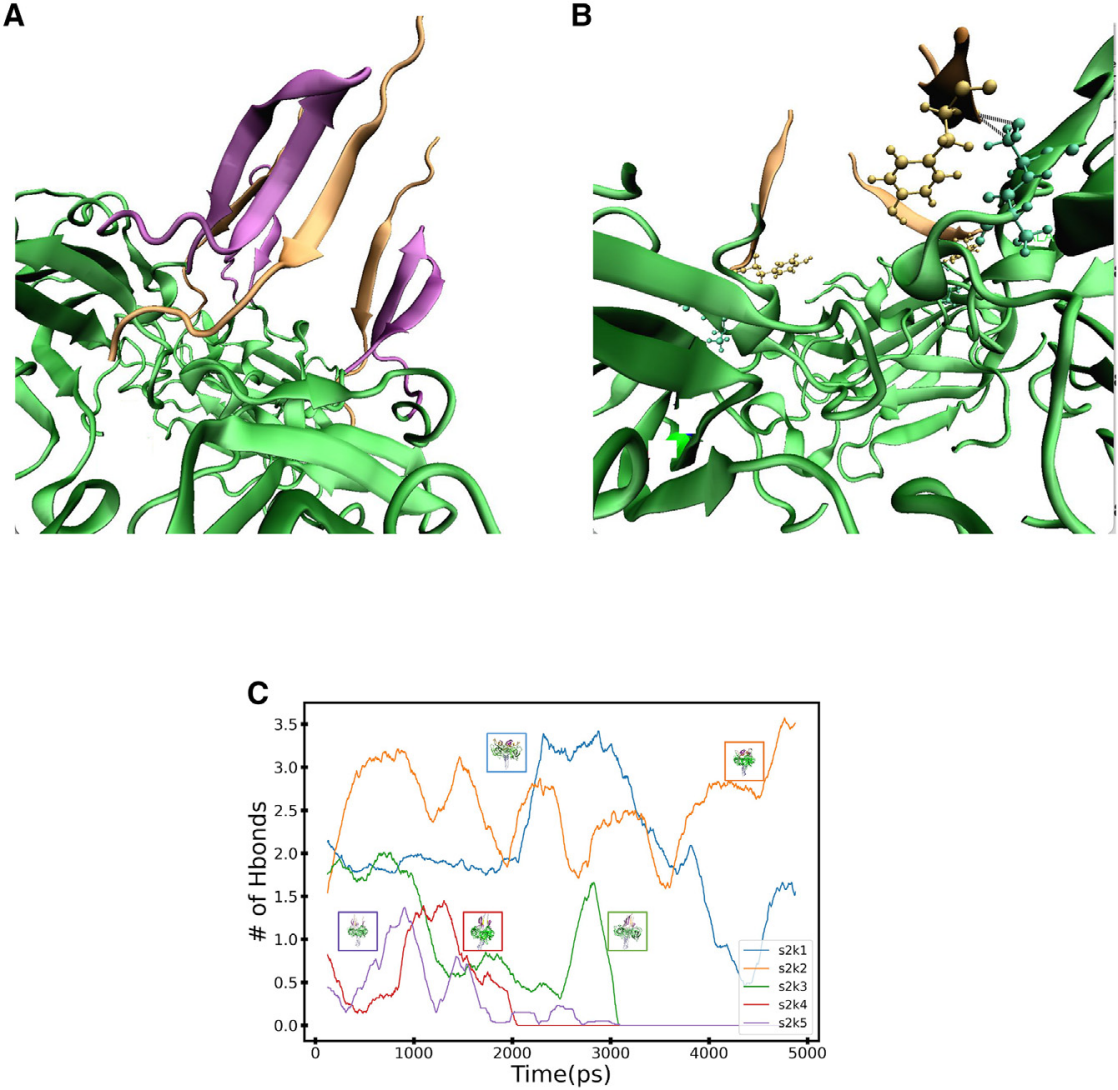
Interactions within 3:3 structures at 1 nm/ns. (*A*) Visual representation of KL (purple, residues 572–577 and 579–584) *β* sheets interacting with KASH (orange). This interaction is the same in all 3:3 structures, especially in SUN2/KASH1,2, where KASH anchors to the SUN through a disulfide bond. The interaction between SUN and KASH is maintained through the *β* sheets. The unfolding of the KL toward the central axis is mediated by the hydrogen bonds between SUN and KASH (*B*). The residues that mediate the unfolding are highlighted in cyan (SUN) and tan (KASH). The hydrogen bond is shown in black dashed lines. The differences between structures is expressed through (*C*) a running average of the number of hydrogen bonds throughout the simulation. Each curve’s color corresponds with the final frame of the structure. SUN2/KASH1,2, structures with disulfide bonds with SUN, never completely lose all of their hydrogen bonds. SUN2/KASH3,4,5 all lose the hydrogen bonds that mediate KL unfolding. The differences are visible in the last frames shown. To see this figure in color, go online.

**Figure 10:**
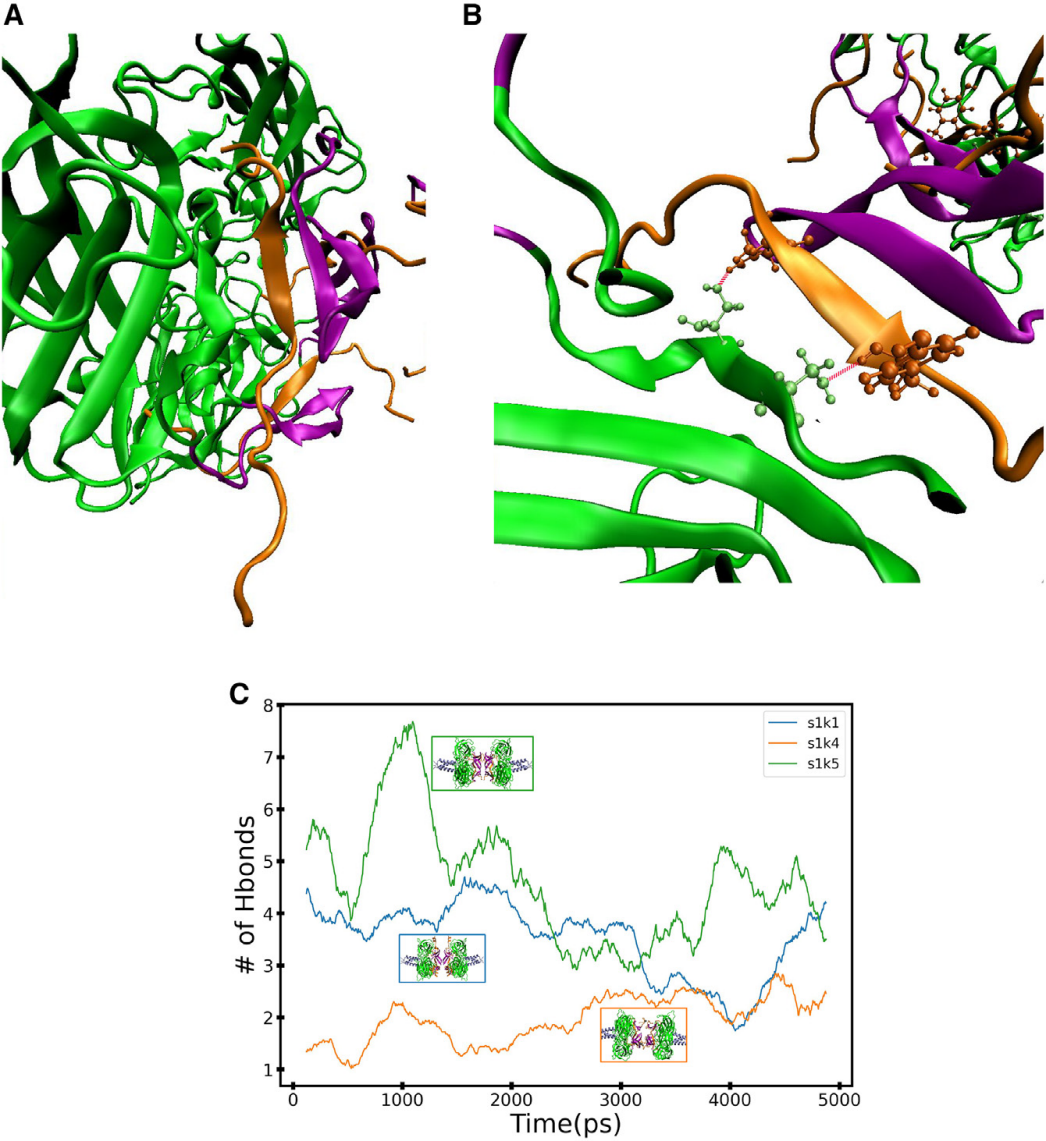
Interactions within 6:6 structures at 1 nm/ns. (*A*) Visual representation of KL (*purple*, residues 666–679) *β* sheets interacting with KASH (*orange*). This interaction is the same in all 6:6 structures. Like the 3:3 model, the interaction between SUN and KASH in the 6:6 model is maintained through the *β* sheets. Unlike the 3:3 structures, the KL in the 6:6 structures does not unfold in the direction of the central axis. We decided to comparatively look at the hydrogen bonds that form between KASH and SUN (*B*). The residues SER651 and SER654 in lime (SUN) form hydrogen bonds (*red*) with the residues in dark orange (KASH). The relationship between the number of hydrogen bonds between these residues over the pulling simulation (*C*) does fall to zero hydrogen bonds. The border color of the last frame of each structure corresponds to the running average curve. To see this figure in color, go online.

To further investigate the changes and differences between the various structures, we looked at other potential anchor points between SUN and KASH. The hydrogen bond that forms between residues 651 in model 6:6, or residue 557 in model 3:3, and KASH serves as a potential structural stabilizer that breaks under loads. Structures that do not have additional anchors between KASH and SUN, such as a disulfide bond, experience a significant drop in the number of hydrogen bonds between the aforementioned SUN residues and KASH (Fig. 9). In Fig. 9 *C*, we can see the difference between structures that lose hydrogen bonding throughout the simulation, where both KASH and KASH-lid are stretched in the same direction as the central axis. This structural change is not observed in the 6:6 structures (Fig. 10). As such, we should not expect the number of hydrogen bonds to drop to zero. Fig. 10 *C* shows all of the structures that maintain hydrogen bonds between residue 651 and/or 654 and KASH. We also expect the structures to maintain their hydrogen bond in the 6:6 structures because of the way the load is applied on KASH.

## Discussion

Recent higher-order assembly models for the LINC complex have debated the putative mechanism of force transfer within the SUN/KASH models. This study provides more evidence for the existence and biological relevance of the higher-order model. Using molecular dynamics simulations, we set out to compare and contrast the putative 3:3 SUN/KASH complex model vs. the new higher-order assembly 6:6 SUN/KASH complex. Here, we discuss the implications of our findings for each model in terms of their structural biomechanics along with their contribution to a broader functional level. We argue that both models can assemble in unique networks and may exist simultaneously in the cell depending on the tasks performed.

Besides anchoring the nucleus, the LINC complex serves as a load bearer and a force transmission agent (33). Within the 3:3 model, the CC region provides the ability to behave elastically as a spring under force. However, the rate at which these forces are applied suggests more complex viscoelastic behavior. This same behavior is not seen in the 6:6 model. Within the 6:6 model under uniaxial pulling simulations, the KASH and the adjacent KASH-lid are not directly being pulled on like the 3:3 model. The elastic behavior that is seen in the 6:6 model is a result of the interactions that maintain the two halves of the 6:6 model. The CC region in model 3:3 and the interface between the halves of the 6:6 model play a significant role in the force transmission mechanism and thereby the stability of the structure.

We showed that SUN/KASH in the 3:3 model and the 6:6 model are force rate dependent (Fig. 8). We also conducted a sample simulation with SUN2/KASH3 at 0.1 nm/ns and obtained consistent results with the 1-nm/ns pulling rate (Fig. S8). Forces are either concentrated and transmitted to the opposite end of the complex or transmitted throughout SUN domain. However, regardless of the pulling rate, the hydrogen-bond analysis shows that the force on KASH is first immediately transferred to the KASH-lid. Our results indicate similar mechanical responses of SUN1 and SUN2 under similar forces. However, it is unclear whether these two proteins are subjected to similar forces in vivo when bound to actin, microtubules, or intermediate filaments. We demonstrated the previous postulates on SUN2/KASH1,2 in the 3:3 model being able to withstand higher forces than other 3:3 structures. Since both KASH1,2 withstand high forces, it is reasonable that KASH1,2 should bind to actin. If we assume that KASH1,2 interacts with SUN in its 3:3 linear form, the structure may serve as a column that maintains the structural rigidity of the nucleus. This may also be why KASH1,2 is found in all cell types. Nesprins 3,4,5, according to the salt bridge and hydrogen-bond analysis, begin to structurally degrade in the 3:3 model under force. Nesprins 3,4,5 are also known to bind to other dynamic cytoskeletal elements, intermediate filaments, and microtubules (16,17,45). The low amount of force these structures can withstand before deformation may also indicate quick cellular processes or may indicate higher-order complexes or large networks. Other parameters, outside of what is discussed, may play a role in the differences observed in the force transmission mechanism of slow and fast pulling rate. We hypothesized that salt bridges between CC and SUN domain as well as within the SUN protomers contribute chiefly to the force mechanism. Our results indicate that they tend to break more easily when forces are applied at a slower rate, leading to the higher fluctuations observed in the structures. When the forces are applied over a shorter time period, these salt bridges do not experience stress immediately and some remain intact for the same displacement. The recruitment of SUN/KASH to an area of the nucleus may still be a mystery, but the cleavage of salt bridges and increased fluctuation suggests that, under a network of SUN/KASH, the fluctuation will decrease. A SUN/KASH cluster would also be force rate dependent like a single unit of SUN/KASH.

The interactions between the *β* sheets of KASH and KASH-lid regardless of the size of KASH or model interact with three residues in all structures. If we accept that KASH-lid is one of the few areas where KASH can interact with SUN, where are the discrepancies? We have shown that SUN2/KASH3,4,5 experience the same amount of force regardless of weight. When the hydrogen bonds between KASH and residue SER557 break, the KASH-lid along with KASH begin to unfold in the direction of the central axis. The disulfide bridge in both SUN2/KASH1,2 serves as an additional anchor point. It also changes the mechanics of pulling. Instead of primarily pulling on the KASH-lid of SUN2/KASH1,2, we are pulling on residue CYS563, which lies between the KASH-lid and the CC region. As KASH is sandwiched between the KASH-lid and the rest of SUN, pulling on KASH in both KASH1,2 structures adds force in the direction of the central axis. However, unlike SUN2/KASH3,4,5, this force is not enough to completely break all the hydrogen bonds formed between KASH and SUN residue SER557.

The same principle of anchoring points can still be applied to the 6:6 structures. The 6:6 structures have more anchoring points, being the head-to-head interaction. Second, the pulling direction is not in the direction of the central axis. These two things cause the effects of forces on 6:6 structures to be more complex. Like SUN2/KASH1,2, having more anchors means the hydrogen bonds between KASH and SUN residue SER651 remain. There is no unfolding of the KASH-lid toward the central axis in the 6:6 structures. Since the pulling direction is not in the direction of the central axis, there is no elongation of the CC region. That difference is also shown in Fig. 8 *B*.

Like the 3:3 model, SUN1/KASH1 from the 6:6 model does not withstand the most force out of all 6:6 structures. Also, unlike the 3:3 model, the salt bridges are not chiefly responsible for the force mechanism. The structures in both 6:6 pulling simulations, transverse pulling, and uniaxial pulling are not and cannot experience the same force on residues as the 3:3 model. The two models are structurally different, and our results show that the structural differences affect the mechanical properties between models. However, like the 3:3 model under slower pulling rates, force is transmitted through the entire structure. This is further exemplified through the RMSF plot in Fig. S6. The slower pulling rate (Fig. S6 *A*) has higher fluctuations than the fast pulling rate (Fig. S6 *B*). The results show that different SUN/KASH complexes respond differently to force. The best way to understand these differences is through the KASH and KASH-lid within the two models. The biggest differences in the higher-order assembly and the putative 3:3 SUN/KASH complex are between complexes that have disulfide bridges and complexes that do not. The disulfide bonds remain an integral part of the structures that we have studied (31,33,46). SUN1/KASH1 also seems like the most probable 6:6 SUN/KASH system to exist in the nuclear envelope. A legitimate concern can be raised about the way SUN1/KASH1 in the 6:6 arrangement handles force if the head-to-head interaction does not hold at larger forces under transverse pulling simulations. KASH1 and KASH2 are meant to interact with actin and other cytoskeletal elements (15,16). Thus, it is reasonable to think that the SUN/KASH will always experience some tension.

Under relatively small loads, SUN1/KASH1 may be in the 6:6 arrangement and, when the cell experiences more loads over time, it undergoes conformational changes to exist in the 3:3 arrangement. These speculations can be cleared up if we had a better understanding of the CC region that spans the perinuclear space. In other higher-order SUN/KASH structures, it has been shown that SUN1 could potentially assemble adjacent to each other instead of the head-to-head interaction that we have shown (44,47,48). This conformational change could potentially happen with SUN1/KASH1 under extreme cellular events such as mitosis or apoptosis (49,50).

The two other 6:6 structures, namely SUN1/KASH4,5, can experience as much force as SUN2/KASH1,2 from the 3:3 model without undergoing as much conformational change as SUN1/KASH1. KASH5 is known to be responsible for meiotic processes, specifically chromosomal movement (51). Although KASH5 is the smallest KASH protein we have observed and KASH4 is slightly larger, it experiences more force under pulling in the 6:6 arrangement; however, in the 3:3 arrangement, SUN2/KASH4,5 experiences considerably less force. This discrepancy suggests that the 3:3 arrangement of SUN2/KASH4,5 must orient in unique clusters to accommodate forces outside of the nuclear envelope. KASH4 is usually expressed in secretory epithelial cells (16). Many studies have shown the importance of zinc in epithelial cells (52). KASH4 also exclusively binds to kinesin-1, which plays a role in nuclear positioning. The 3:3 arrangement of SUN2/KASH4 does not contain any zinc ions in the crystal structure; however, the 6:6 arrangement of SUN1/KASH4 has zinc as part of its structure. There may be some mediated action that facilitates the recruitment and potentially releases zinc from SUN/KASH to perform specific tasks. It is possible that, under conditions where the concentration of zinc ions in the perinuclear space is high, KASH4 in the 3:3 arrangement may undergo a conformational change that results in the 6:6 arrangement of KASH4. The lack of conformational change leads us to assume how SUN1/KASH4,5 orientates itself in the nucleus in higher-order networks or SUN/KASH clusters.

**Figure 11:**
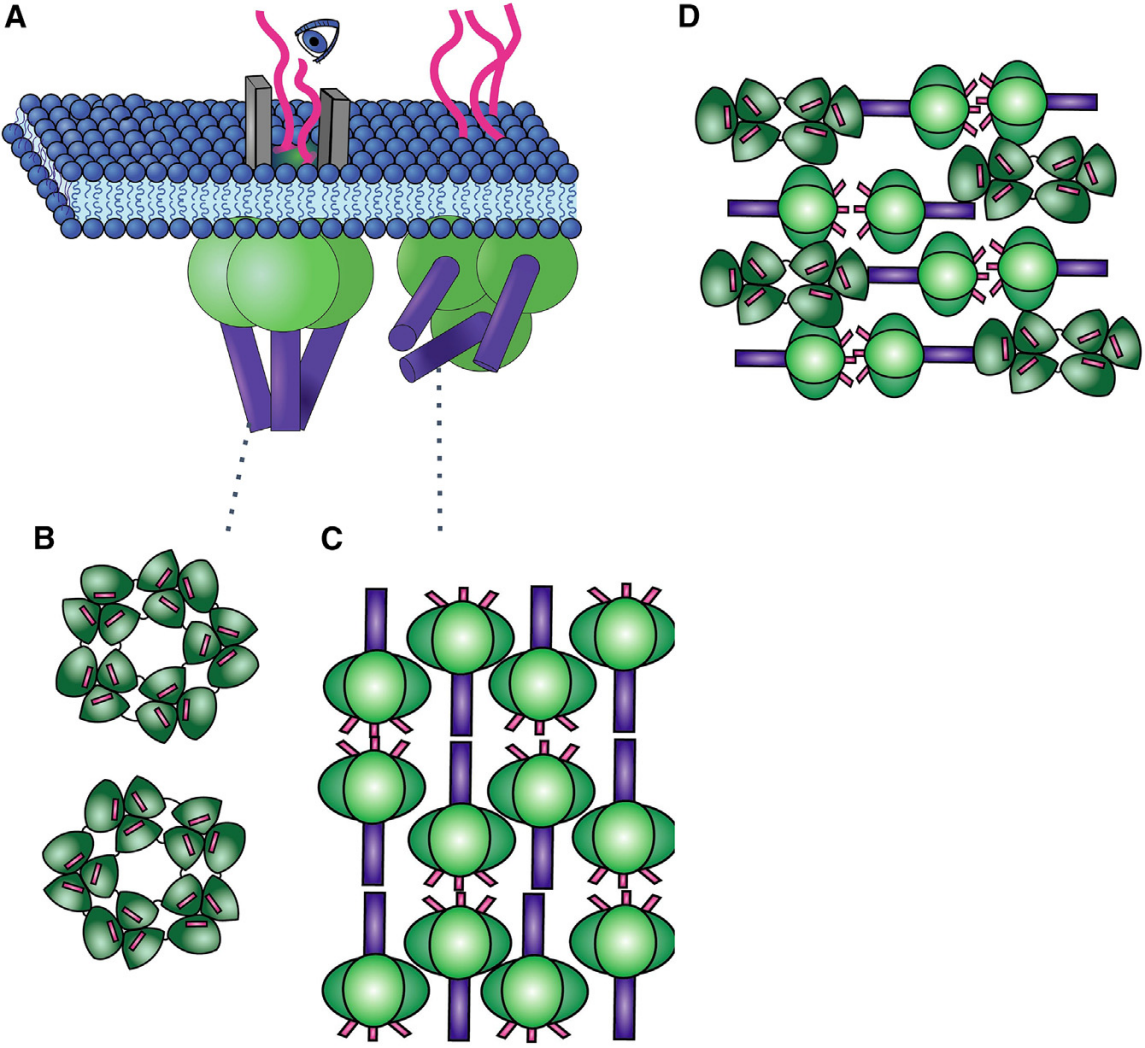
Proposed network of higher-order clustering of different SUN/KASH complexes. (*A*) An orthographic cartoon depicting the potential ways both 3:3 structures and 6:6 structures may associate in the outer nuclear envelope. The KASH (*pink*) extends from the outer nuclear envelope (*blue*), whereas SUN sits on the underside. (*B*) A top-view depiction of how the 3:3 SUN/KASH complexes form a unique star-shaped network. (*C*) A representation of a top view of 6:6 SUN/KASH structure interacting to form a matrix network. (*D*) A representation of how both 3:3 and 6:6 structures come together to form a uniquely packed matrix network. To see this figure in color, go online.

As there is not as much of a conformational change seen in the SUN1/KASH4,5, one may wonder how these structures localize in the nuclear membrane and what network SUN and KASH make in the nuclear envelope. Even though we have the crystal structure of SUN/KASH, we do not have a clear understanding of the CC region that spans the perinuclear space. Since the 6:6 KASH4 and KASH5 do not undergo any drastic conformational changes, it would be unrealistic for the CC region to bend 90° without other protein elements. Different arrangements of SUN/KASH can be postulated in the nucleus, as depicted in Fig. 11. These SUN/KASH arrangements cluster to form unique symmetries that can span the nuclear envelope. Both 3:3 and 6:6 arrangements cluster in various configurations (Fig. 11 *B* and *C*). As previously stated, we recognize that both 3:3 and 6:6 arrangements can exist in biologically relevant conditions simultaneously in the nucleus; therefore, a hybrid version of both arrangements is also conceivable in the nuclear envelope (Fig. 11 *D*). Thus, it would be likely that there exist elements along the SUN CC region that add to the mechanics of SUN/KASH and contribute to a greater meshwork of the nuclear envelope. These networks may add to the mechanical characteristics of the nucleus.

One thing that we considered is the potential effect a change in pH would cause the structures. It is possible that changes in pH can significantly perturb the stability of this complex. Within this context, two crucial aspects emerge, significantly influencing the mechanical behavior of the SUN/KASH system. First, altering pH levels could profoundly affect various facets of structural stability and the assembly of the SUN/KASH complex. Notably, prior research has demonstrated the profound influence of pH on the formation of disulfide bonds, a vital component of SUN structures. Specifically, all SUN structures feature disulfide bridges within the SUN/KASH complex, and, in the case of SUN1/KASH1 and SUN2/KASH1,2, disulfide bonds connect the SUN and KASH proteins. A shift in pH conditions unfavorable for disulfide bond formation could significantly affect the transmission of forces across these structures. Additionally, changes in pH have the potential to disrupt the stability of salt bridges present within the SUN/KASH structures. Such disruptions would invariably alter the way mechanical forces are transmitted throughout the SUN/KASH complex. Hence, we recognize that pH fluctuations have the potential to exert a profound influence on the mechanical behavior and overall stability of the SUN/KASH complex, a factor worthy of careful consideration in future investigations.

The force required to pull apart Apo-SUN2 is considerably smaller than all other SUN/KASH complexes (Fig. S5). This may functionally suggest a few things. First, Apo-SUN2 may have a special recruitment pathway where both maintaining the head-to-head interaction and detaching from the head-to-head are important for switching between the two arrangements. The ability to attach and detach also could play a role in nuclear membrane localization. Finally, it may imply how important KASH is for distributing and withstanding higher forces.

## Conclusions

We have shown the importance of different pulling rates and directions in the mechanobiology of the LINC complex. The central question of this study was to determine what SUN/KASH arrangement is more likely to exist in the cell and how these complexes may cluster in the nucleus. We believe that both arrangements may exist simultaneously depending on the type of cells, the stage of the cell cycle, and the functions performed. The linear trimer model may be suitable for fast tensile force transmission and high loads, whereas the higher-order assembly model may be suitable for describing the flexibility of the nuclear envelope. We also suggest that, when these clusters form, they augment and amplify the structural rigidity and dynamics of the nuclear envelope. Nonetheless, we do not have a clear understanding of the other components that may be involved in the force transfer mechanisms, especially in the case of SUN1/KASH1. These binding partners of the LINC complex should be investigated to better grasp the extent of their influence on the arrangement of the SUN/KASH complexes.

## Author contributions

Z.J. and M.R.K.M. designed the study. G.Y., N.D., and J.O. performed the simulations. G.Y. and N.D. analyzed and interpreted the results. G.Y. and N.D. drafted the manuscript. G.Y., N.D., Z.J., and M.R.K.M. revised the manuscript.

## Supporting material

Document S1. Figures S1–S8

Document S2. Article plus supporting material
