# Supplementary material for: Force transmission and SUN-KASH higher-order assembly in the LINC complex models
Source: Biophys J. 2023 Nov 2;122(23):4582–97. doi: 10.1016/j.bpj.2023.11.001 (PMC10719071; doi:10.1016/j.bpj.2023.11.001)
Supplement: Document S1. Figures S1–S8 [file mmc1.pdf]

Biophysical Journal, Volume *122*

Supplemental information

Force transmission and SUN-KASH higher-order assembly in the LINC complex models

Ghafar Yerima, Nya Domkam, Jessica Ornowski, Zeinab Jahed, and Mohammad R.K. Mofrad

Supporting Material

Force transmission and SUN-KASH higher-order assembly in the LINC complex models

Ghafar Yerima[1△], Nya Domkam[1△], Jessica Ornowski[1], Zeinab Jahed[2,3,*], Mohammad R.K. Mofrad[1,4,*]

[1] Molecular Cell Biomechanics Laboratory, Departments of Bioengineering and Mechanical Engineering, University of California, Berkeley, CA 94720, USA.

[2] Department of Chemistry, Stanford University, CA 94305, USA

[3] Department of Nanoengineering, Jacobs school of Engineering, University of California, San Diego, CA 92039, USA

[4] Molecular Biophysics and Integrative Bioimaging Division, Lawrence Berkeley National Lab, Berkeley, CA 94720, USA.

△ These authors contributed equally to this work

*Co-corresponding Authors mofrad@berkeley.edu, zjahed@ucsd.edu

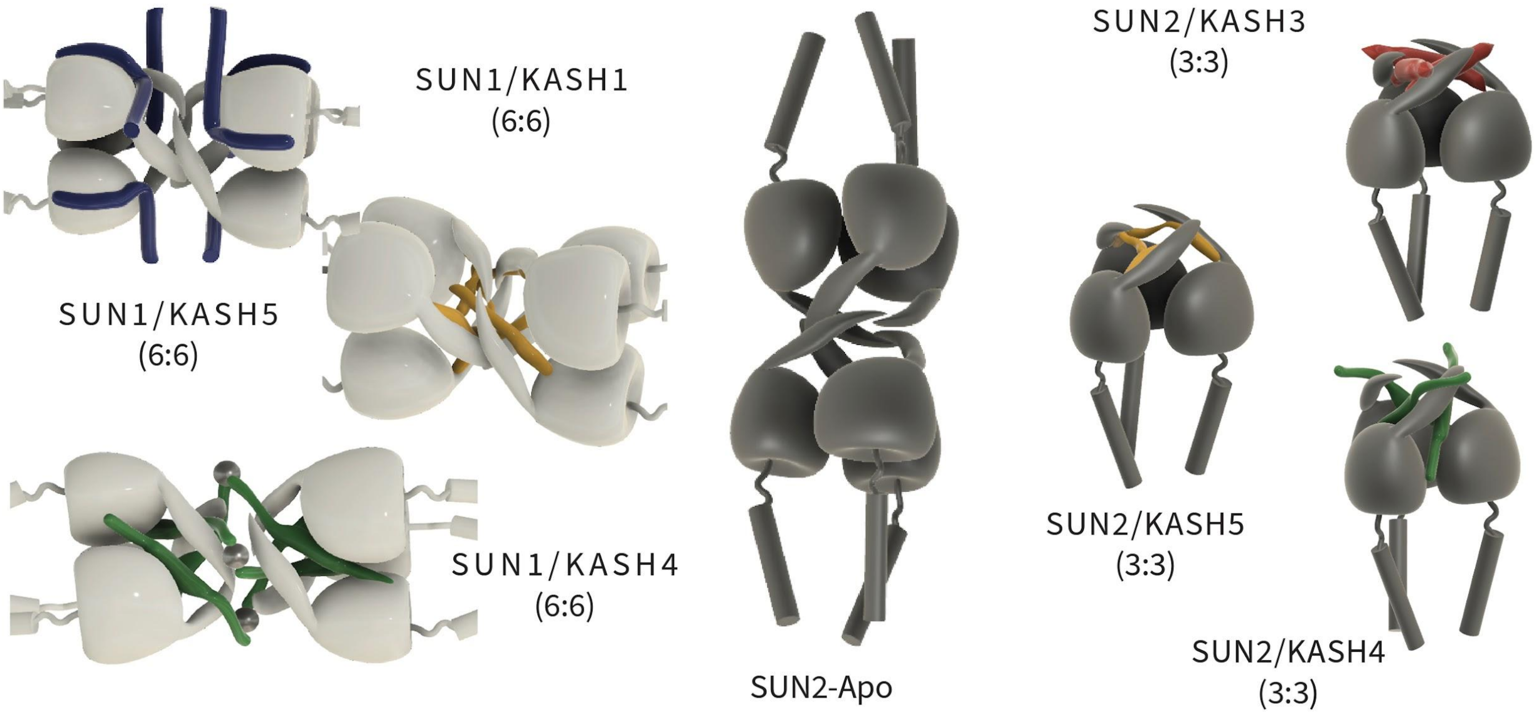

Figure S1: 3D rendition of various SUN/KASH models.

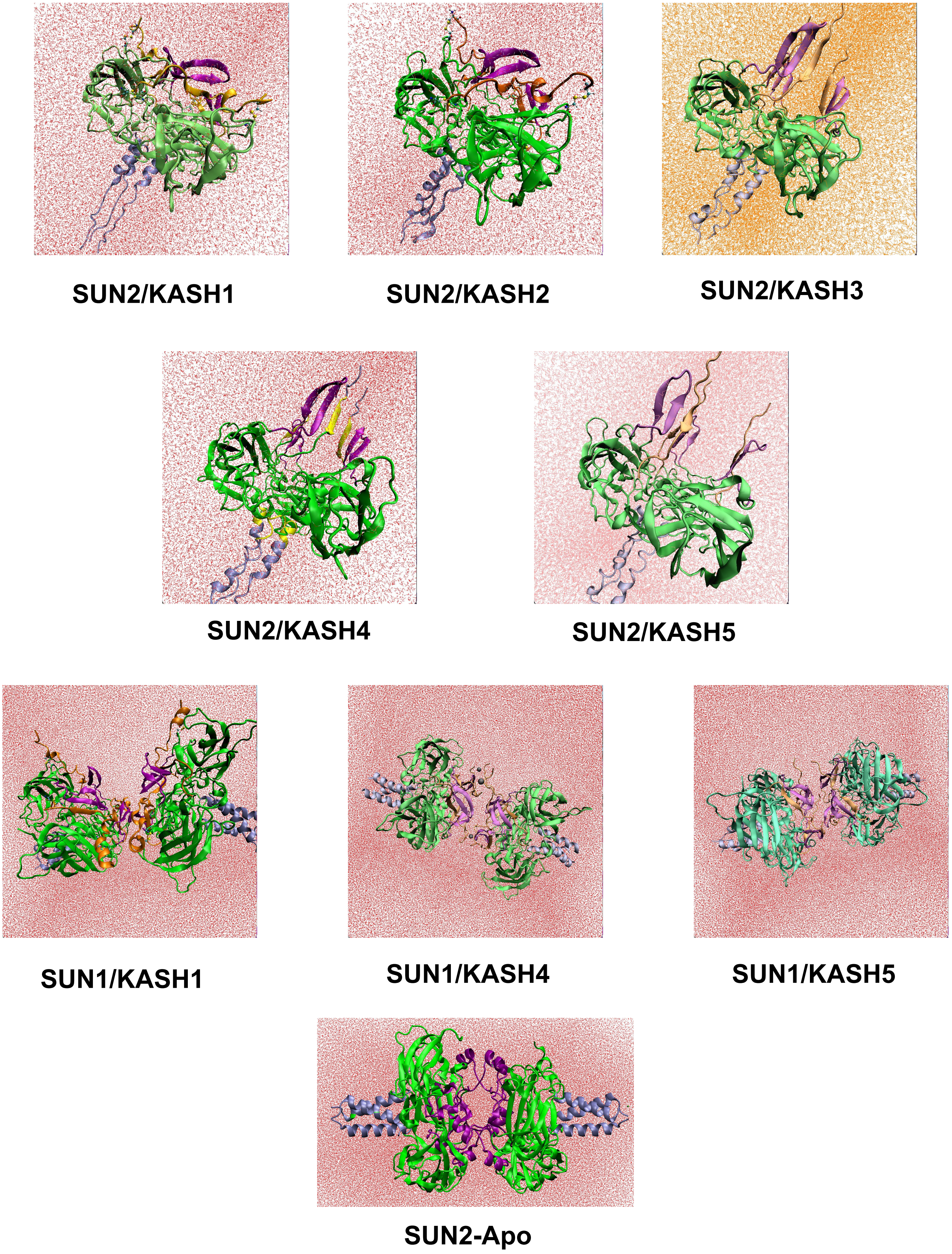

Figure S2: Atomistic models of SUN/KASH complexes in water.

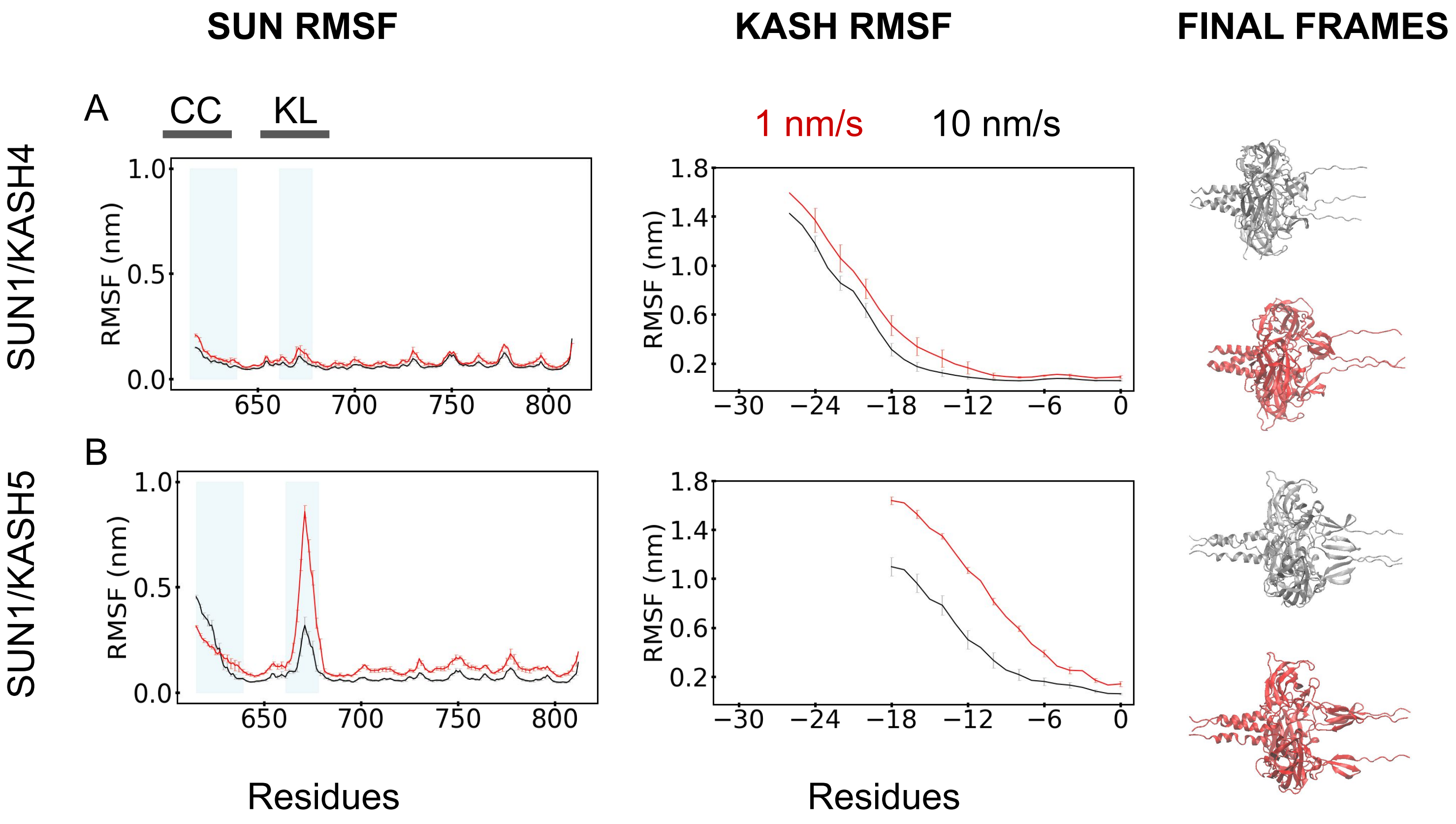

Figure S3: Rate dependent force response of SUN1 in complex with KASH4,5. Root Mean Square Fluctuation (RMSF) of SUN1 for **A)** KASH4, **B)** KASH5 are shown in the SUN RMSF (left) column. RMSF of the KASH proteins is shown in the KASH RMSF (middle) column. The red and black curves represent the 1 and 10 nm/ns pulling rates respectively. The x-axis for the SUN RMSF column graphs ranges from 522 to 716 according to the SUN2 domain residue numbering. The x-axis for the KASH RMSF column graph ranges from -24 to 0 following a sequence alignment based numbering of KASH proteins. **CC** represents the coiled-coil domains and **KL** represents the KASH-lids. These two areas are shaded in blue on the SUN column graphs. For each graph, the data for 3 simulations were averaged. For each simulation, the data for all protomers were averaged. Both pulling rates reach the same displacement of 5 nm.The final frames of the 10 (silver) and 1 (red) nm/ns pulling rate simulations are shown for each structure in the right column.

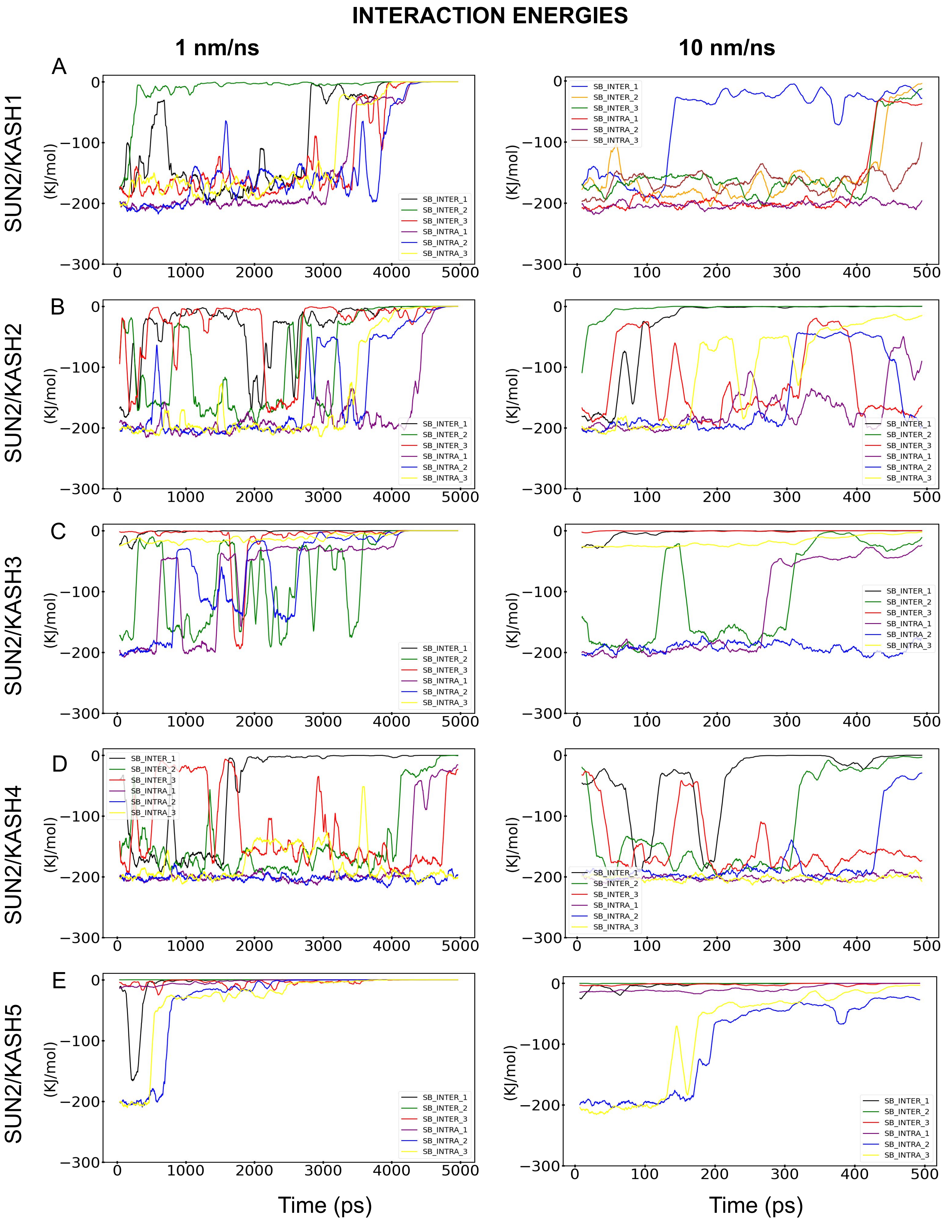

INTERACTION ENERGIES
1 nm/ns
10 nm/ns
A
B
C
D
E
SUN2/KASH1
SUN2/KASH2
SUN2/KASH3
SUN2/KASH4
SUN2/KASH5
(KJ/mol)
Time (ps)
SB_INTER_1
SB_INTER_2
SB_INTER_3
SB_INTRA_1
SB_INTRA_2
SB_INTRA_3

Figure S4: Interaction energies between salt bridges in SUN2 structures. Short-range coulombic interaction energies of SUN2 in complex with **A)** KASH1, **B)** KASH2, **C)** KASH3, **D)** KASH4, **E)** KASH5 are shown for the 1 nm/ns rate (left column) and 10 nm/ns (right column). SB-inter 1-3 denotes the three intermolecular salt bridge pairs in each structure and the SB-intra 1-3 shows the three intramolecular salt bridges. The x-axis displays the simulation time in ps, while the y-axis shows the energy in kJ/mol. For each structure and pulling rate, only one sample simulation energy is shown.

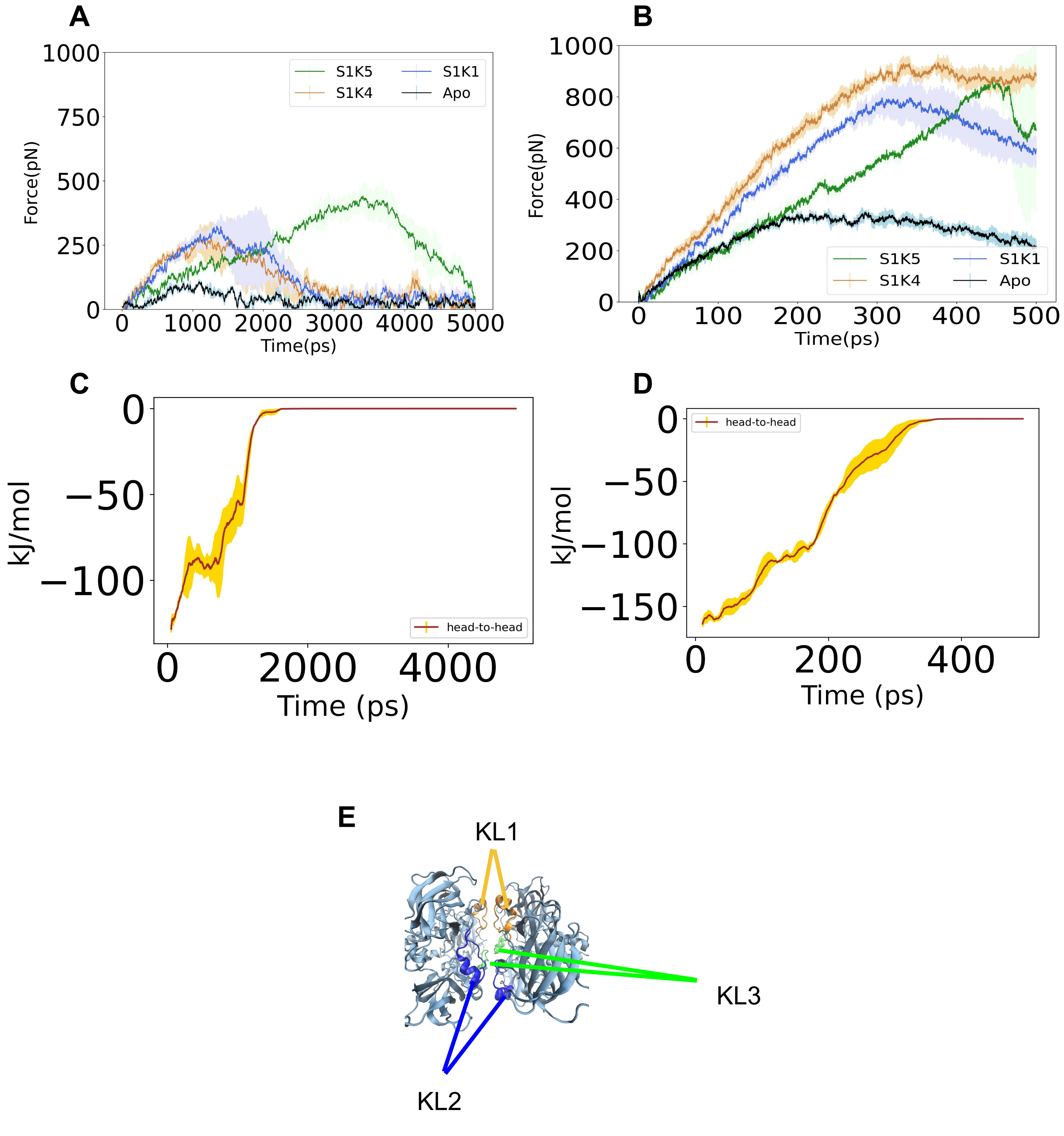

Figure S5: Both **A** and **B** show the uniaxial pulling force in different pulling rates, 1 nm/ns and 10 nm/ns, respectively. SUN2 Apo structure under slow pulling experiences less force over a shorter time before it dissociates. The the structures respond to different pulling rates which is not dependent on length. **C** and **D** are looking at the head-to-head interaction energy for different pulling rates,1 nm/ns and 10 nm/ns, respectively, over three simulations. **E** is diagram showing the different KASH lid pairs.

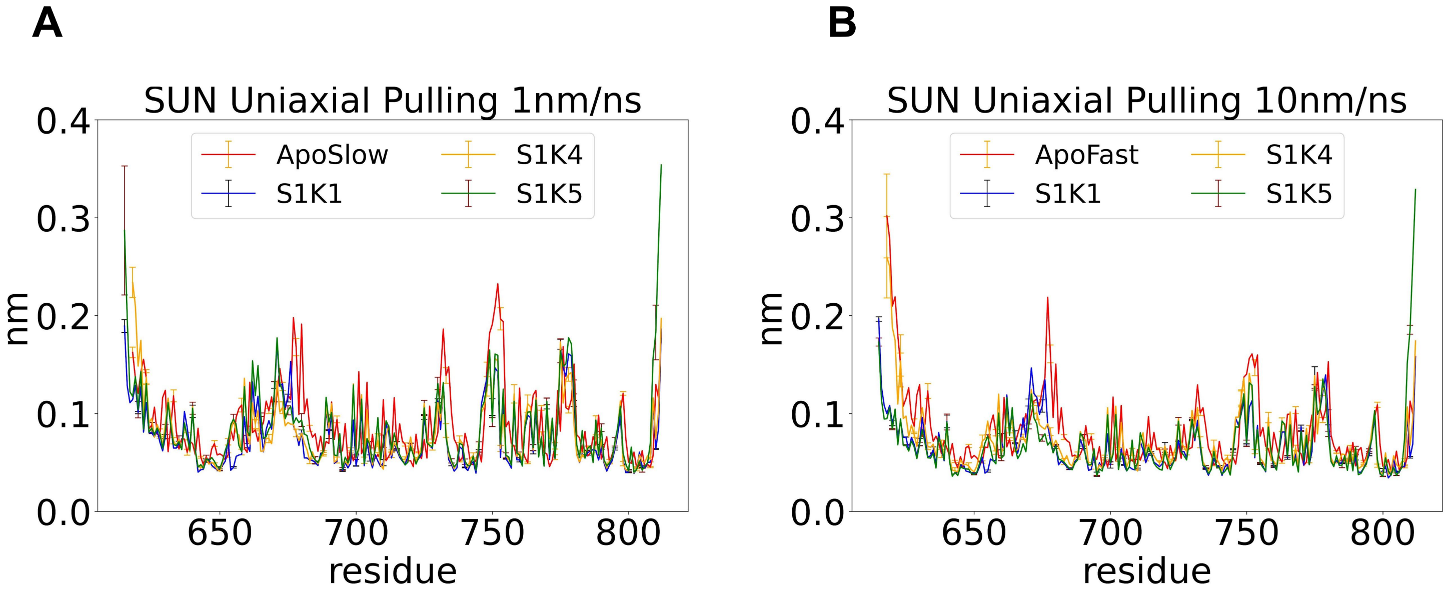

Figure S6: RMSF of Apo-SUN2 and other 6:6 structures. **A)** and **B)** show the RMSF for uniaxial pulling in different pulling rates, 1 nm/ns and 10 nm/ns, respectively.

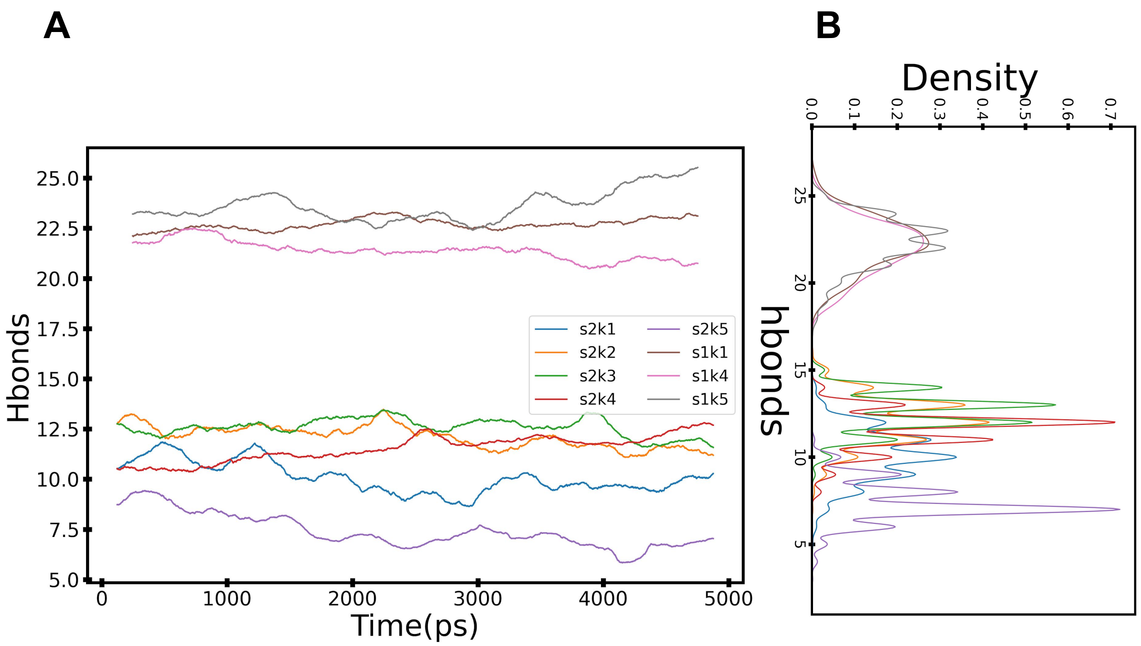

Figure S7: Hydrogen bonds analysis **A)** Plot of hydrogen bonds over the simulation time between **KASH residues** 8787 to 8791 (SUN1/KASH1), 8786 to 8791 (SUN2/KASH1), 6874 to 6878 (SUN2/KASH2), 964 to 970 (SUN2/KASH3), 393 to 398 (SUN1/KASH4), 393 to 398 (SUN2/KASH4), 553 to 557 (SUN1/KASH5), 788 to 792 (SUN2/KASH5) and **KASH-lid residues** 666 to 679 (SUN1) or residues 572 to 577 and 579 to 584 (SUN2). The 6:6 structures cluster with the same hydrogen bonds throughout the simulation. The 3:3 structures are similar with the exception of SUN2 KASH5. **(B)** Kernel Density Estimation (KDE) plot of the number of hydrogen bonds of beta sheets between 3:3 structures tend to center around 10 to 12 hydrogen bonds with the exception of KASH5. The reasoning behind this, is within the crystal structure SUN2/KASH5. The KASH lid in this structure is missing a residue in the KASH lid region, causing a slight shift in the KDE plot. The curves of the 6:6 plots have more hydrogen bonds because there are twice as many KASH-lid/KASH pairs. The number of hydrogen bond changes over the simulation time. The hydrogen bonds over the simulation time for all structures does not vary more than 2 hydrogen bonds.

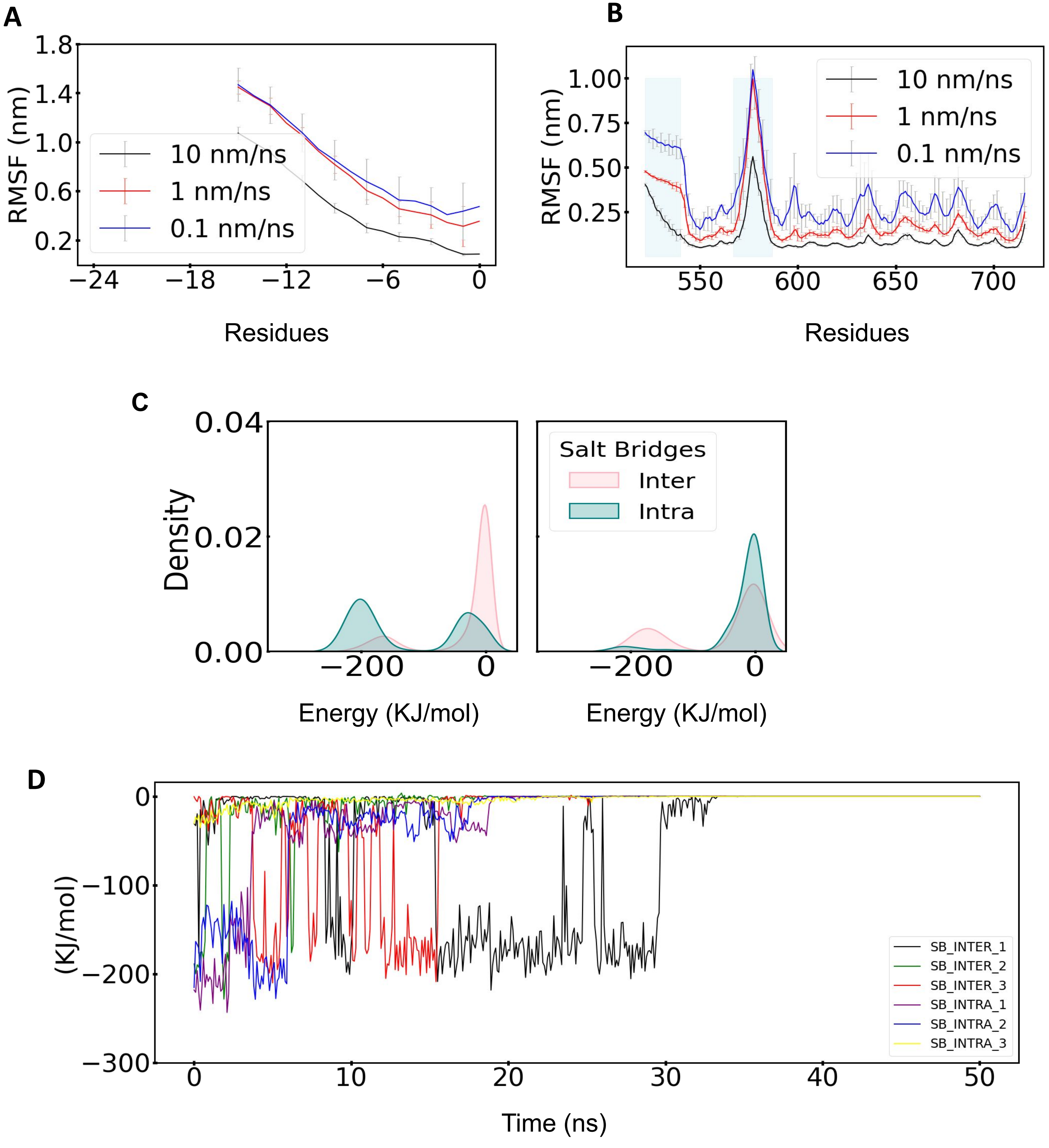

Figure S8: Comparison of SUN2/KASH3 structural dynamics under different pulling rates. **A)** RMSF analysis of KASH3 reveals consistent fluctuations under both 1 nm/ns and 0.1 nm/ns pulling rates. However, the 10 nm/ns pulling rate has a noticeably lower fluctuation compared to the other slower pulling rates. The same behavior is observed in **B)** the RMSF of SUN2 at three different pulling rates. While the KASH-lid region displays similar fluctuations under 1 nm/ns and 0.1 nm/ns pulling rates, other regions of SUN2 experience increased fluctuation at the slowest pulling rate of 0.1 nm/ns. **C)** Density plots of the interaction energies between inter and intra salt bridges for 1 nm/ns (left) and 0.1 nm/ns (right). **D)** Interaction energies over simulation time for 0.1 nm/ns.
